# Antidiabetic and Antihyperlipidemic Activities and Molecular Mechanisms of *Phyllanthus emblica* L. Extract in Mice on a High-Fat Diet

**DOI:** 10.3390/cimb46090623

**Published:** 2024-09-20

**Authors:** Hsing-Yi Lin, Cheng-Hsiu Lin, Yueh-Hsiung Kuo, Chun-Ching Shih

**Affiliations:** 1 Department of Internal Medicine, Cheng Ching Hospital, No. 139, Pingdeng St., Central District, Taichung City 40045, Taiwan; 2 Department of Internal Medicine, Fengyuan Hospital, Ministry of Health and Welfare, Fengyuan District, Taichung City 42055, Taiwan; 3 Department of Chinese Pharmaceutical Sciences and Chinese Medicine Resources, China Medical University, Taichung City 40402, Taiwan; 4 Department of Nursing, College of Nursing, Central Taiwan University of Science and Technology, No. 666 Buzih Road, Beitun District, Taichung City 40601, Taiwan

**Keywords:** insulin resistance, *Phyllanthus emblica* L., antihyperlipidemic

## Abstract

We planned to explore the protective activities of extract of *Phyllanthus emblica* L. (EPE) on insulin resistance and metabolic disorders including hyperlipidemia, visceral obesity, and renal dysfunction in high-fat diet (HFD)-progressed T2DM mice. Mice treatments included 7 weeks of HFD induction followed by EPE, fenofibrate (Feno), or metformin (Metf) treatment daily for another 4-week HFD in HFD-fed mice. Finally, we harvested blood to analyze some tests on circulating glycemia and blood lipid levels. Western blotting analysis was performed on target gene expressions in peripheral tissues. The present findings indicated that EPE treatment reversed the HFD-induced increases in blood glucose, glycosylated HbA1_C_, and insulin levels. Our findings proved that treatment with EPE in HFD mice effectively controls hyperglycemia and hyperinsulinemia. Our results showed that EPE reduced blood lipid levels, including a reduction in blood triglyceride (TG), total cholesterol (TC), and free fatty acid (FFA); moreover, EPE reduced blood leptin levels and enhanced adiponectin concentrations. EPE treatment in HFD mice reduced BUN and creatinine in both blood and urine and lowered albumin levels in urine; moreover, EPE decreased circulating concentrations of inflammatory NLR family pyrin domain containing 3 (NLRP3) and kidney injury molecule-1 (KIM-1). These results indicated that EPE displayed antihyperglycemic and antihyperlipidemic activities but alleviated renal dysfunction in HFD mice. The histology examinations indicated that EPE treatment decreased adipose hypertrophy and hepatic ballooning, thus contributing to amelioration of lipid accumulation. EPE treatment decreased visceral fat amounts and led to improved systemic insulin resistance. For target gene expression levels, EPE enhanced AMP-activated protein kinase (AMPK) phosphorylation expressions both in livers and skeletal muscles and elevated the muscular membrane glucose transporter 4 (GLUT4) expressions. Treatment with EPE reduced hepatic glucose-6-phosphatase (G6Pase) and phosphoenolpyruvate carboxykinase (PEPCK) expressions to suppress glucose production in the livers and decreased phosphorylation of glycogen synthase kinase 3β (GSK3β) expressions to affect hepatic glycogen synthesis, thus convergently contributing to an antidiabetic effect and improving insulin resistance. The mechanism of the antihyperlipidemic activity of EPE involved a decrease in the hepatic phosphorylation of mammalian target of rapamycin complex C1 (mTORC1) and p70 S6 kinase 1 (S6K1) expressions to improve insulin resistance but also a reduction in hepatic sterol regulatory element binding protein (SREBP)-1c expressions, and suppression of ACC activity, thus resulting in the decreased fatty acid synthesis but elevated hepatic peroxisome proliferator-activated receptor (PPAR) α and SREBP-2 expressions, resulting in lowering TG and TC concentrations. Our results demonstrated that EPE improves insulin resistance and ameliorates hyperlipidemia in HFD mice.

## 1. Introduction

The 10th revised statistical epidemiological analysis of the International Diabetes Federation prophesied that the incidence of diabetes mellitus is estimated to reach 783 million by 2045 [1] and will therefore increase the economic burden of patient health care worldwide. Type 2 diabetes mellitus (T2DM) is one of the metabolic disorders associated with either β-cell dysfunction or insulin insensitivity in peripheral tissues and is categorized as insulin resistance [2], which is a main part of metabolic syndromes [2]. The basic symptomatology of metabolic syndromes includes hyperinsulinemia, dyslipidemia, insulin resistance, and abdominal obesity [3]. Metabolic disorders in T2DM include a reduction in the sugar transport ability but a rise in hepatic glucose production. Thus, management strategies that enhance glucose uptake while decreasing gluconeogenesis are important in the treatment of T2DM [3].

Previous evidence demonstrated that glucose transporter 4 (GLUT4) exhibited a key part in keeping circulating euglycemia [4]. In addition to insulin stimulation, muscular contraction promotes glucose uptake shifting GLUT4 translocation toward cell membrane [5]. Skeletal muscular expressions of insulin-induced GLUT4 translocation in T2DM individuals were found to be significantly reduced [6]. Thus, the enhancement in GLUT4 levels or induction of translocation could provide a potential drug for development to treat diabetes.

There are key pathways in T2DM that are prompted by insulin or contraction, meaning that the promoting glucose uptake to skeletal muscles includes mechanisms of insulin-dependent inducing Akt/PKB activity or contraction-controlled motivation [3,7], and hypoxia-controlled activation of AMP-activated protein kinase (AMPK) [3,8]. AMPK plays a target in the remedy of T2DM [3]. AMPK is important in glycemic and lipid metabolism. Evidence suggests that T2DM is prone to lipid and glucose catabolism dysregulation, and therefore an AMPK activator is an encouraging remedy [9].

Metformin has been shown to cause AMPK activation and facilitate muscular GLUT4 translocation resulting in insulin-independent sugar uptake [10]. Metformin is prescribed in the management of T2DM and decreases glycemia concentrations mainly by reducing sugar production in livers but enhancing skeletal glucose uptake [9]. In the liver, glucose-6-phosphatase (G6Pase) and phosphoenolpyruvate carboxykinase (PEPCK) function as rate-restricting enzymes in controlling gluconeogenesis [11]. Metformin activates hepatocyte-specific AMPK, thus leading to decreased acetyl-CoA carboxylase (ACC) activity and elevated fatty acid (FA) oxidation, but inhibition of expressions of lipogenic enzymes [9].

The transcription factor forkhead box protein O1 (FOXO1) is demonstrated to play a core part in interfering with insulin activity on gluconeogenesis in the liver [12]. Both AMPK phosphorylation and insulin are known to suppress G6Pase and PEPCK transcription [13]. Blocking FOXO1 offers an opportunity for novel therapies for diabetes mellitus [12].

Akt (PKB) can stimulate glucose uptake and synthesis of glycogen using affecting GLUT4 [14] and glycogen synthase kinase 3β (GSK3β) [12]. GSK3β influences insulin resistance and was phosphorylated but inhibited by Akt [15]. Akt and GSK3β activity is mutually controlled in insulin resistance [15].

Fenofibrate is a fibrate that functions as a ligand for peroxisome proliferator-activated receptor α (PPARα), which downregulates several genes and controls lipid metabolism [16]. Fenofibrate is a prescription drug for the management of hypertriglyceridemia [17].

Fatty acid synthase (FAS) is an important enzyme in relation to fatty acid synthesis [18]. Sterol regulatory element binding proteins (SREBP-1c and -2) modulate target expressions in relation to lipogenesis and cholesterol biosynthesis [19]. SREBP1c is a critical modulator of fatty acid synthesis, while SREBP2 chiefly regulates cholesterol synthesis [20,21]. PPARα functions as the controller of β-oxidation in the livers [22]. PPARγ plays the core modulator of adipocyte differentiation and stimulates adipogenesis [23].

Enhanced mammalian target of rapamycin (mTOR) signaling is demonstrated in pathologies, including T2DM and obesity [24,25,26,27]. mTORC1 raises lipid synthesis and protein synthesis, which responses partly occur via the mTOR substrate phosphorylation, such as ribosomal S kinase 1 (S6K1) [24,25,26,27].

**Figure 1:**
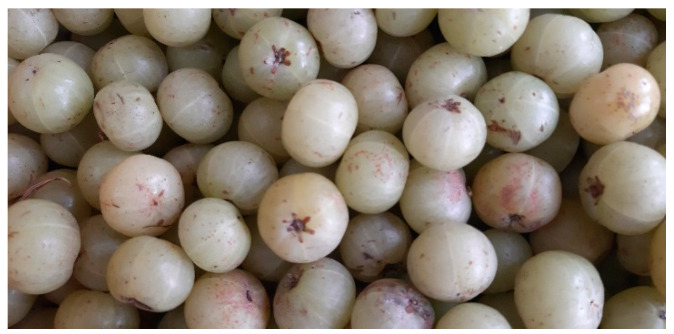
The fruits of *Phyllanthus emblica* L.

*Phyllanthus emblica* L. (Figure 1), known as the Indian gooseberry, belongs to the family Euphorbiaceae and is the most important medicinal agent in the traditional Indian system of medicine. Their fruits were beneficial functional activities for individuals [28,29,30,31]. For example, evidence showed that the ellagic acid and gallic components of these fruits exhibit the functional activities of hepatoprotection and antioxidants [32]. Currently, we examine the functional activities and mechanisms of an extract of *P. emblica* and demonstrate that *P. emblica* extract exhibits a favorable activity on immunoregulation and hypoglycemia in two non-obese diabetes (NOD)-induced T1DM mouse models [33] and in STZ-T1DM mouse model [34]. *P. emblica* is rich in polyphenols that exhibit antioxidant activity. Evidence has shown that *P. emblica* displays an antioxidant activity [28,29,31,32]. The fruits are used to treat ailments including diabetes, antitussive [28], liver complaints, inflammation, cardiac disorders, jaundice, and cataractogenesis [35]. Some of the recent in vitro studies revealed anti-inflammatory [36], nephrotoxicity modulation [37], and hepatoprotective [38] properties.

A high-fat diet (HFD) fed to C57BL/6J mice exhibits insulin resistance, hyperlipidemia, obesity, or higher levels of blood FFA [39]. This special species of mouse fed with HFD for a long period progressed to obesity, diabetes mellitus, and renal dysfunction marked by proteinuria and the increase in blood creatinine and urea nitrogen (BUN) [40]. Elevated plasma free fatty acid (FFA) level is due to enhanced adipose lipolysis results from an increase in adipose mass or other reasons [41].

The HFD feeding in the long-term, which contributes to obesity and develops metabolic disorders, could modify the lipid metabolism within the kidney and result in kidney injury in the mouse models [40,42].

Previous evidence has shown that the inflammation cytokine plays an important part in the progress of diabetic nephropathy [43]. Urinary albumin excretion implies the meaning of kidney dysfunction [44]. Thus, this model of HFD-induced mice was employed in this study. Based on research indicating that AMPK activation modulates various metabolic disorders, it was acknowledged as the target in the management of metabolic syndromes, such as T2DM and hyperlipidemia [45,46]. The Thr^172^ of α subunits phosphorylation plays a vital role in AMPK activation [47].

According to functions of GLUT4 and AMPK phosphorylation in relation to glucose uptake and metabolism described above, we investigated whether EPE treatment could exert glycemic control activities and further pursued the mechanism of EPE on the skeletal muscular membrane GLUT4 and AMPK phosphorylation expressions in HFD mice and compared it with the clinical antidiabetic and antihyperlipidemic drugs metformin (Metf) or fenofibrate (Feno). The regulated gene expressions associated with antidiabetic mechanisms (including hepatic PEPCK and G6Pase) were also investigated. The skeletal muscle has been shown to be the main location of whole-body insulin-interceded glucose uptake [48]. There are three aspects to this research. We are going to start by examining how EPE could modulate target gene expressions in C2C12 myotube, then assess the preventive activities by HFD-fed mice, and finally, follow with exploring how seven re-fractions of EPE affect the target AMPK phosphorylation expressions in C2C12 myotubes.

## 2. Materials and Methods

### 2.1. Chemicals

Antibodies against GLUT4 (no. sc-79838) were purchased from Santa Cruz Biotechnology, Inc. (Dallas, TX, USA). PPARγ (no. ab45036), p-AMPK (Thr^172^), and PPARα (no. ab8934) antibodies were purchased from Abcam Inc. (Cambridge, MA, USA). Antibodies to p-Akt (Ser^473^) (no. 4060), FAS (no. 3180), t-AMPK (Thr^172^), PEPCK1 (D12F5) rabbit mAb (no. 12940S), acetyl-CoA carboxylase (C83B10) rabbit mAb (no. 3676), phospho-mammalian target of rapamycin (mTOR) (Ser^2481^) antibody (no. 2974), β-actin (no. 4970), and total mTORC1 (no. 2972) were obtained from Cell Signaling Technology Inc. (Beverly, MA, USA). G6Pase antibody (NBP1-80533) and SREBP2 antibody (NB100-74543) were from Novus Biologicals (E Easter Ave, Centennial, CO, USA). SREBP-1C antibodies (p-Ser^439^) (LS-C368524) were purchased from Lifespan Bioscience (Seattle, WA, USA).

### 2.2. Ethyl Acetate Extract of P. emblica L. (EPE) Preparation

Fruits of *P. emblica* L. (Figure 1) are obtained from Miaoli County, Ogan Marketing Cooperation, Taiwan. This part performance including preparation of fruit extract and seven re-fractions of EPE) was as previously described [33,34].

### 2.3. Cell Culture and Target Gene Expression in C2C12 Myoblasts

This part adapted C2C12 skeletal myoblasts (ATCC, CRL-1772) to perform the target gene expression estimation as in a previous report [49,50,51,52].

### 2.4. Animals, Diets, and Grouping

These research studies were conducted and proceeded based on the regulations of the Institutional Animal Care and Use Committee of the university (no. 110-CTUST-001). Fifty-six C57BL/6J male mice (four-week-old) were purchased from the National Laboratory Animal Breeding Center (Taipei, Taiwan). All mice were randomly parted into seven groups as follows: control group (CON) comprising control diet (no: Diet 12450B from Research Diets, Inc., New Brunswick, NJ, USA) meaning low-fat diet (LD) (10% fat) (*n* = 8); six groups comprising HFD (no: Diet 12451) (45% fat) as previous studies [52,53]. The six groups of HFD mice were daily fed with 45% HFD throughout the experiment for 11 weeks. Following the 7 weeks induction of LD or HFD, the HFD mice (*n* = 48) had re-separated into 6 groups (*n* = 8, each set) as the followings: three groups treated with extract of *P. emblica* (EPE) comprising a low concentration of EPE: 100 mg/kg/day body weight (bw), a middle concentration (200 mg/kg/day bw), and a high concentration (400 mg/kg/day bw) of EPE, and these were groups named EPE1, EPE2, or EPE3, respectively; positive control group 1: fenofibrate (Feno: 250 mg/kg/day bw); positive control group 2: metformin (300 mg/kg/day bw); and these six groups of mice daily treated with vehicle (the high-fat control (HF) group), EPE1, EPE2, EPE3, Feno, or Metf for 4 weeks (by oral gavage) [52].

The control/treatment groups are presented as follows:

CON: Control group; control diet: low-fat diet (LD) (10% fat);

HF: type 2 diabetic Control; HFD (45% fat);

HFD+ EPE1: type 2 diabetic +Treatment 1 (100 mg/kg);

HFD+ EPE2: type 2 diabetic +Treatment 2 (200 mg/kg);

HFD+ EPE3: type 2 diabetic +Treatment 3 (400 mg/kg);

HFD+ Feno: type 2 diabetic + comparative drug 1 (anti-hyperlipidemic drug);

HFD+ Metf: type 2 diabetic + comparative drug 2 (anti-diabetic drug).

For example, the dosage of Feno is 250 mg/kg bw, and the preparation of Feno solution, calculation, and determination of choice concentrations in the Feno-treated group were described in the following. To make the solution of dosage 250 mg/body weight of Feno, for 100 g body weight mice, 25 mg of Feno and vehicle (ex: carboxymethylcellulose; CMC) were added to make the total volume of the solution up to 1 mL, that is, 2.5 g Feno was dissolved in 0.5% CMC to make the 100 mL total volume of the solution. Whenever administering Feno according to the body weight of the mice on that day (if body weight is 30 g), we administered 0.3 mL Feno to the mice.

Calculation and preparation of the solution of dosage Feno (250 mg/g body weight):

25 mg/100 g body weight → dissolved in 1 mL solvent, that is,

25:1 = X mg:100 mL,

X = 25 × 100 (mg),

X = 2500/1000 (g),

X = 2.5 (g), that is,

2.5 g Feno → dissolved in solvent (ex: CMC) to make up the 100 mL total volume of the solution.

Feno and Metf was dissolved in CMC solvent.

After treatment with EPE, Feno, or Metf for 28 days, mice were sacrificed after a 12-h fast. The peripheral tissues were weighed, and stored at −80 °C. The 24-h urine was harvested to assess renal function. In addition to blood glucose levels, the results of this study involved the improvement of T2DM, such as biomarkers, and adipocytokine were examined as previously reported [50,53]. The time of daily drug treatment was about AM 8:30~9:30. After treatment with EPE, Feno, or Metf for 28 days, the mice were sacrificed. The night (about PM 8:30~9:30) before sacrifice, food was removed, and mice fasted for 12 h. During the procedure, a 100 μL blood sample was collected from the retro-orbital sinus of mice and for blood glucose level analysis. On the day of sacrifice, 0.6–0.8 mL blood sample was collected from the posterior vena cava of mice, and then put in the heparin tube, followed by mixture, centrifugation at 3300 rpm for 10 min, and then the adapted 0.3–0.4 mL plasma for later analysis. After treatment with drugs, a single mouse was put in a metabolic cab, and a 24-h urine sample was collected for analyses of renal function.

#### 2.4.1. Blood Glucose and Biochemical Markers Monitoring

After treatments, we collected blood samples (the 12-h fasting) from the retro-orbital sinus to examine blood glucose concentrations. This part was performed (including blood percent HbA1c, plasma TC, TG, adiponectin, and leptin concentrations) as in previous studies [49,52,53].

#### 2.4.2. Morphological Analysis

Histological hepatocellular ballooning findings’ designation comprises grade 0, none; grade 1, mild; grade 2, moderate; and grade 3, severe based on a previous study [54]. The examination of adipose and liver specimens by a microscope, camera, and Image Software version 28 was described in previous studies [49,52,55].

#### 2.4.3. Estimation of Inflammation Cytokine and Renal Function

This part was performed including CRP, KIM-1, NALP3/NLRP3, BUN, and creatinine as in the previous study [55].

### 2.5. Western Blotting Assessment

The immunoblot procedures for the assessment of muscular membrane GLUT4, t-AMPK (Thr^172^), p-AMPK (Thr^172^), hepatic PPARα and FAS, adipose PPARγ and FAS expressions, and the analysis procedure including secondary antibody, Enhanced Chemiluminescent (ECL) were as previously described [49,52,55].

### 2.6. Assessment of p-AMPK/t-AMPK Expressions of the Re-Fractions of EPE in C2C12 Myotube

Since AMPK activation is a critical part of molecular mechanisms of T2DM and hyperlipidemia, cell culture analysis of AMPK phosphorylation was performed to clarify the antidiabetic activity in T2DM by Western blotting test. The administration of cells with insulin or re-fractions of EPE (EA-1~EA-7) was described in previous studies [33,53]. The seven re-fractions preparation was described as in previous studies [33].

### 2.7. HPLC and Determination of Phenolic Compounds

This part was performed as written in previous studies [33,55]. Our previous findings have shown that the EPE contains ellagic acid, chebulagic acid, and gallic acid as previously described [33,34,55].

### 2.8. Statistics

All data were presented as a mean and standard error coupling with ANOVA and Dunnett’s test. A *p*-value less than 0.05 is presented as a significant difference.

## 3. Results

### 3.1. GLUT4 Expression and AMPK and Akt Activation in C2C12 Myotubes

**Figure 2:**
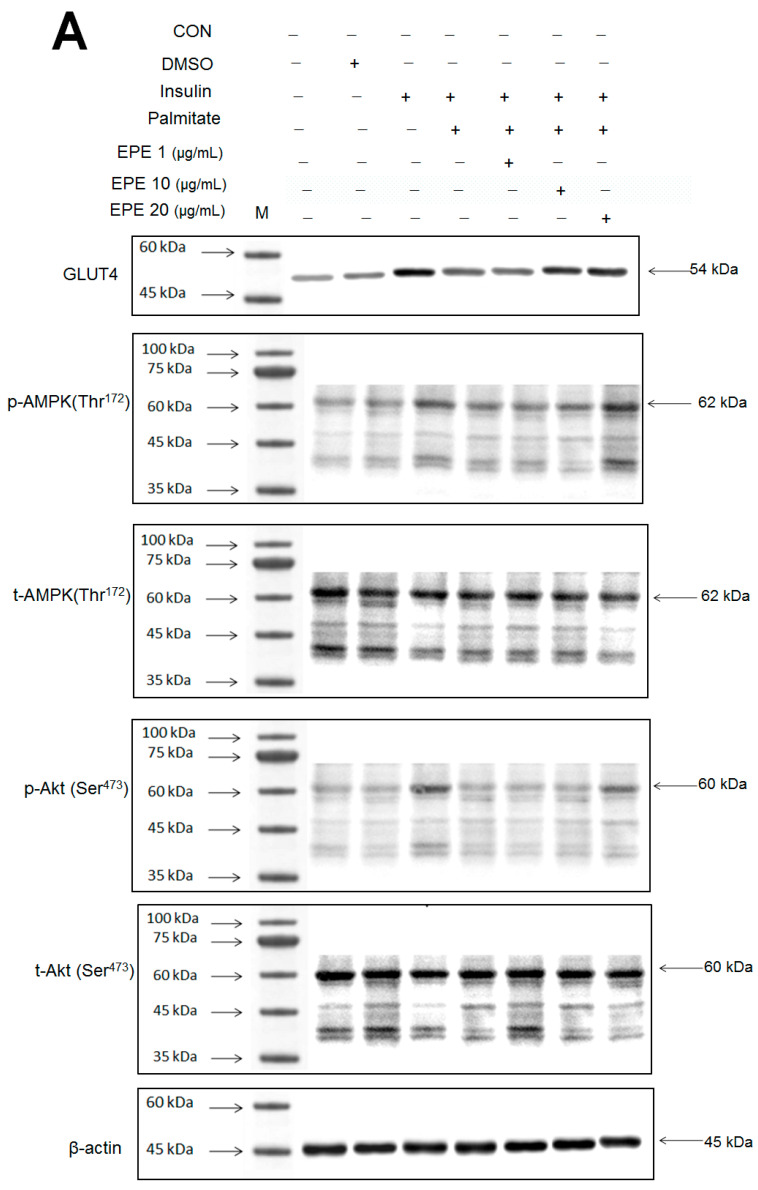

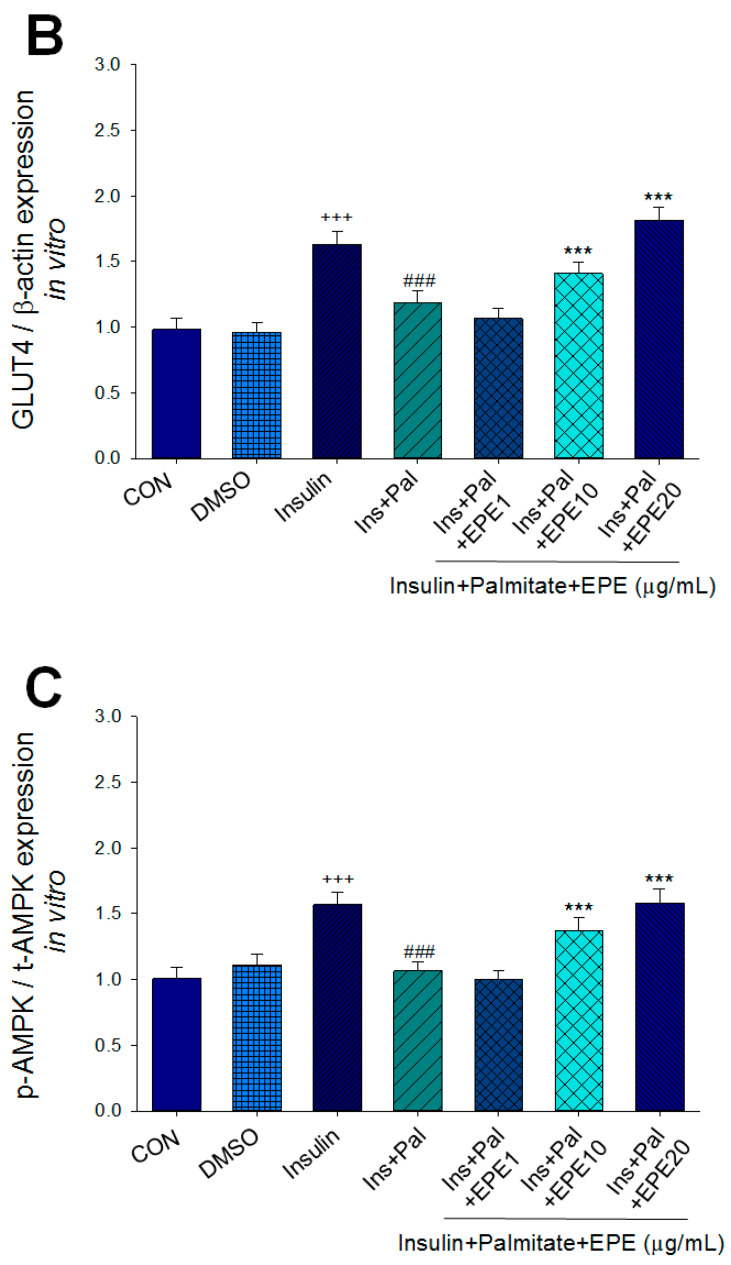

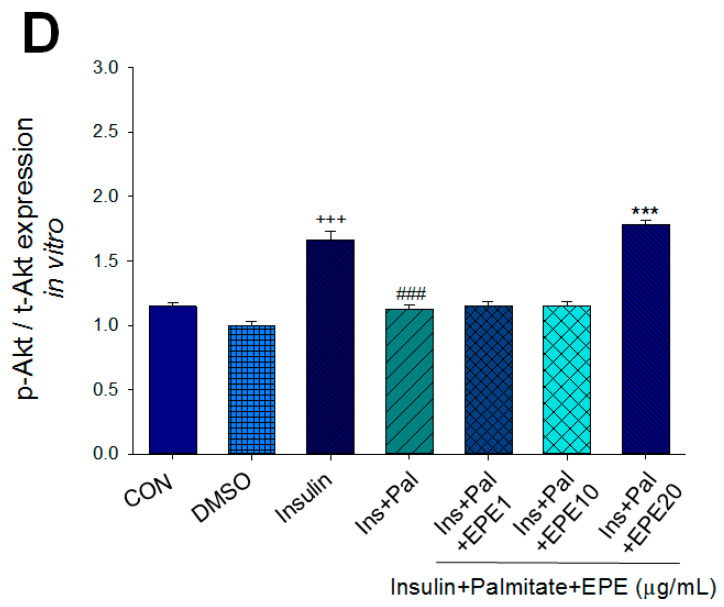
Effects of ethyl acetate extract of *Phyllanthus emblica* L. (EPE) on the insulin (Ins)-stimulated expression levels of membrane glucose transporter type 4 (GLUT4), the ratio of phospho-5′-adenosine monophosphate kinase (p-AMPK) to total AMPK (t-AMPK), or phospho-Akt (p-Akt)/total Akt (t-Akt) in insulin-resistant C2C12 myotube cells induced by palmitate (Pal). The symbols “+++”, “###” and “***” represent *p* < 0.001 as, respectively, compared to the value of the blank control, positive control (insulin) and negative control (insulin + palmitate) using analysis of variance (ANOVA) and with Dunnett’s tests. (**A**) Representative image, (**B**–**D**) quantification of the membrane GLUT4 expression levels, the ratio of p-AMPK to t-AMPK, or p-Akt/t-Akt expression levels. CON, the blank control; DMSO, a solvent control. All values are means ± SE (*n* = 3).

Figure 2A–D showed that the membrane GLUT4 expressions in vitro were markedly reduced by palmitate; this reduction was reversed by 5, 10, and 25 μg/mL EPE. The expressions of AMPK activation in vitro were dramatically reduced by palmitate; this reduction was reversed by EPE at 5, 10, or 25 μg/mL. The p-Akt/t-Akt expressions in vitro were markedly reduced by palmitate; this reduction was reversed at 25 μg/mL EPE.

### 3.2. Animal Treatments

#### 3.2.1. Body Weight, Weight Gain, Liver Weights, and White Adipose Weights

**Figure 3:**
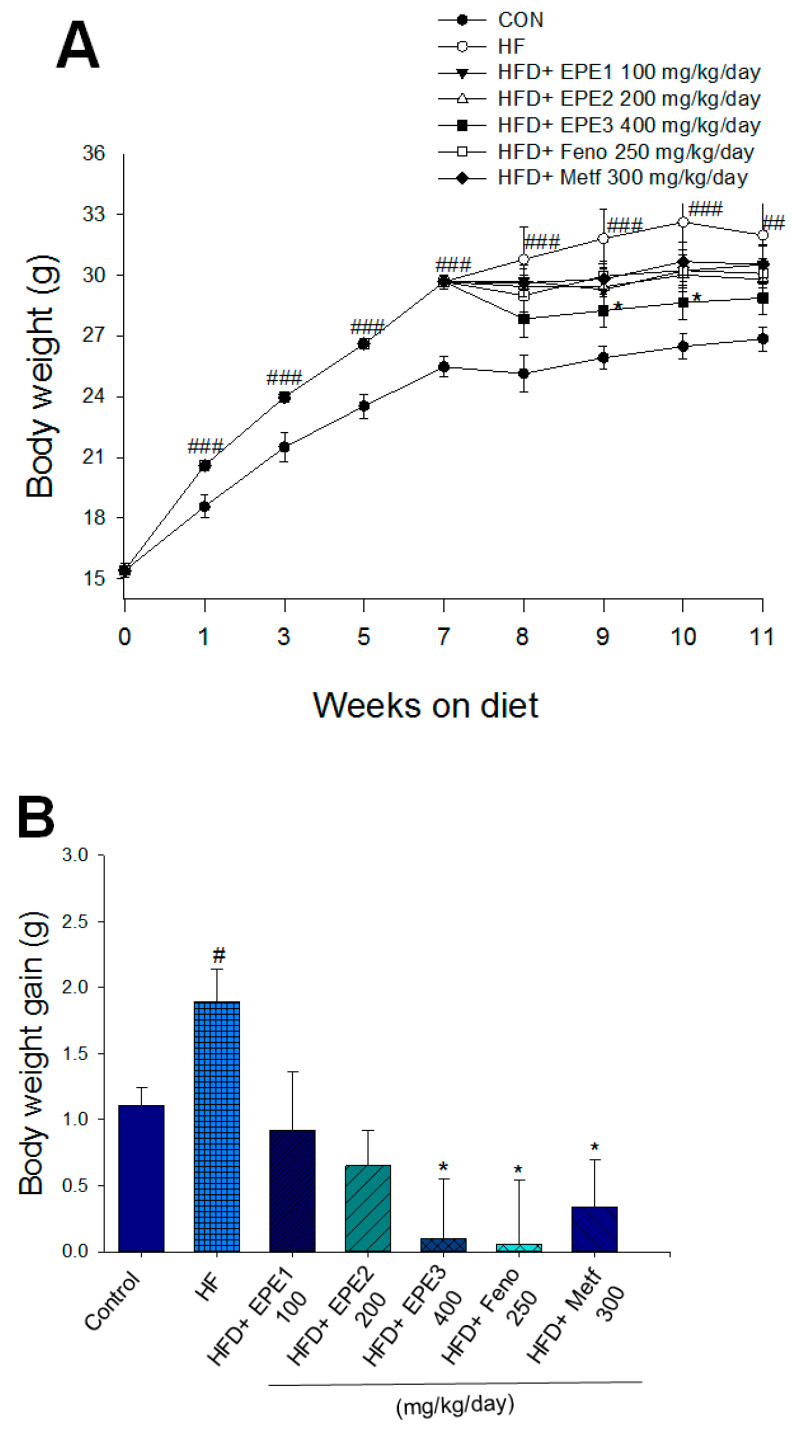

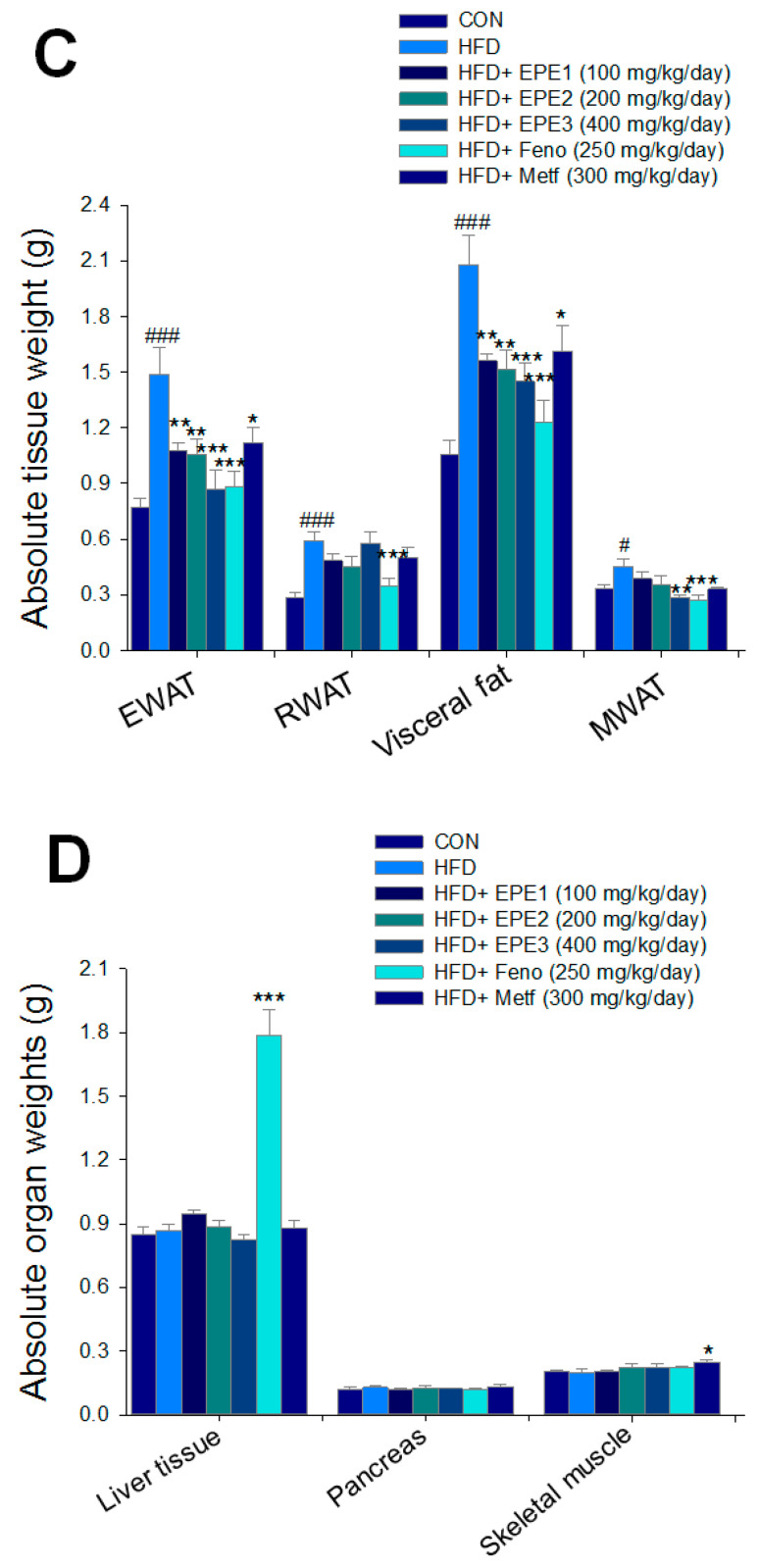

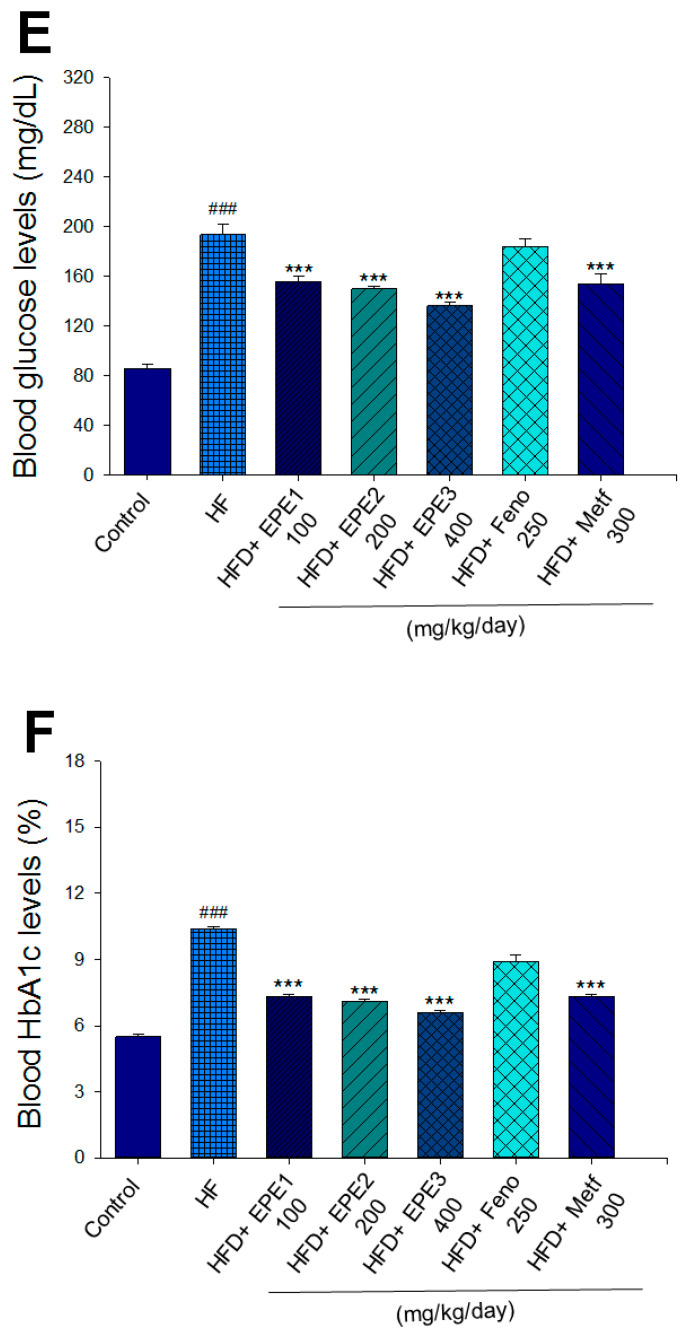

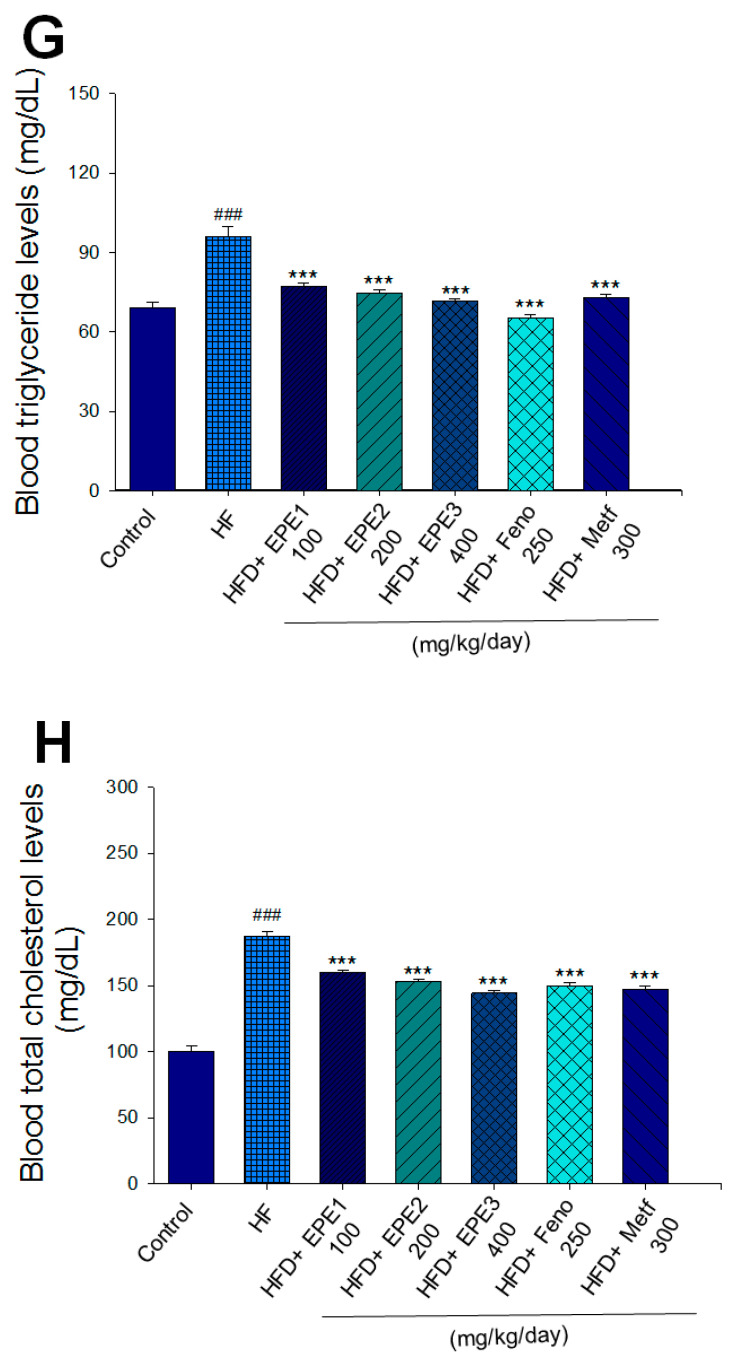

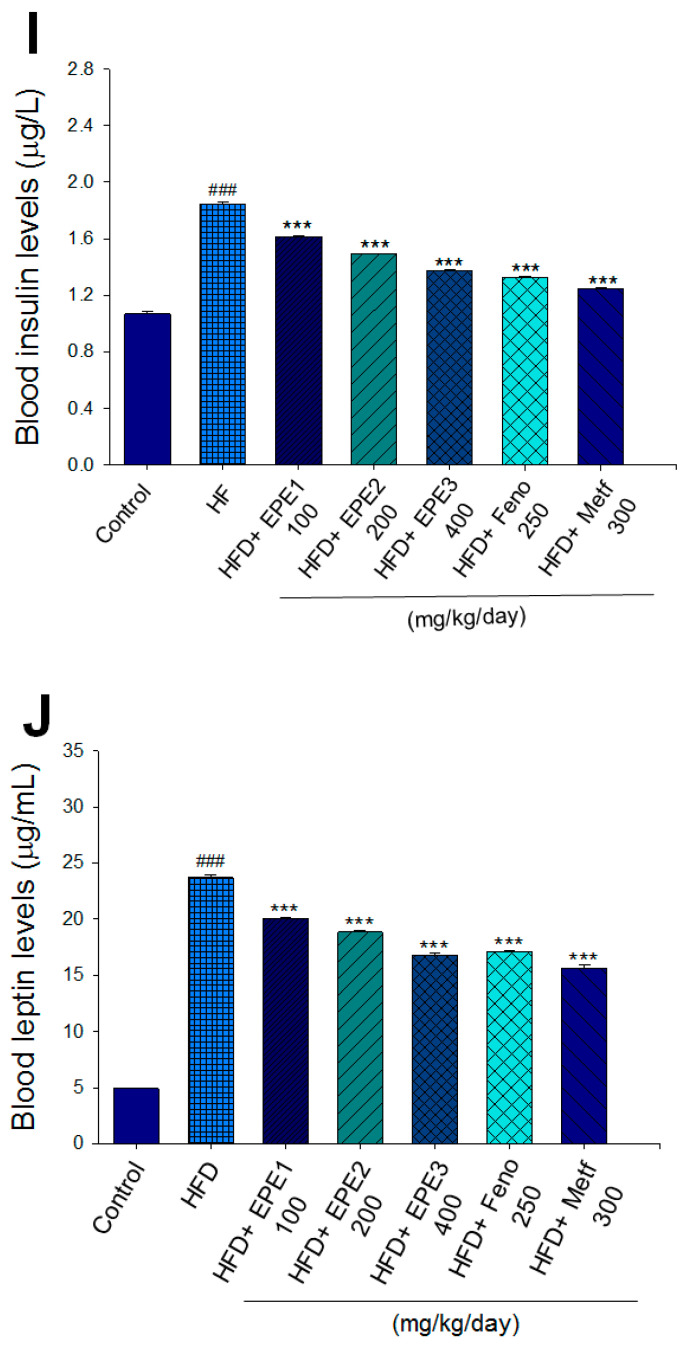

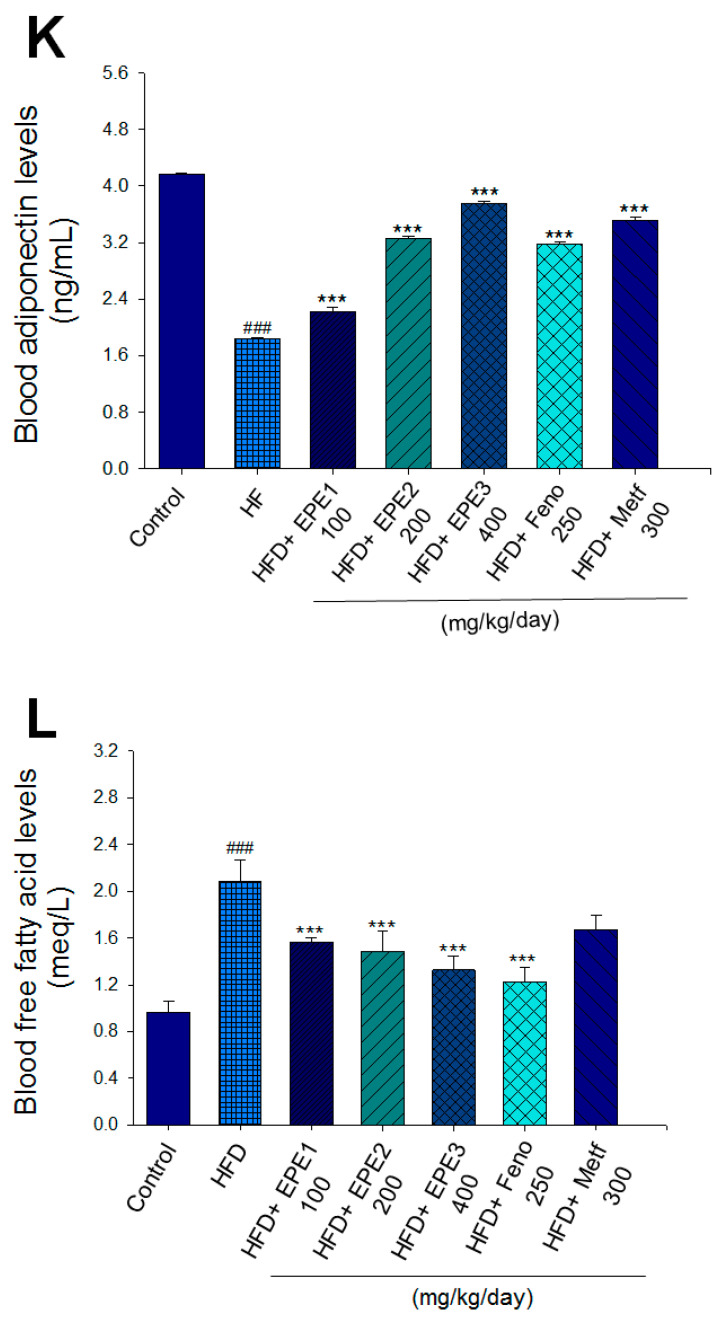

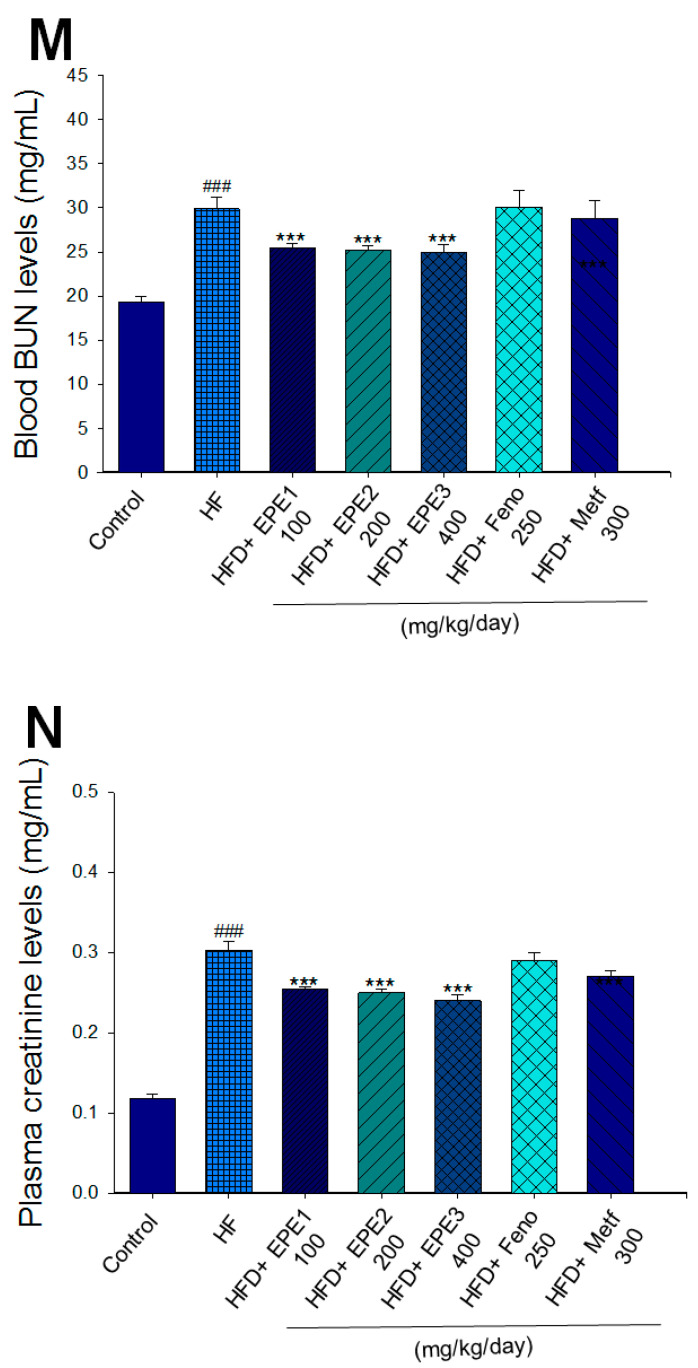

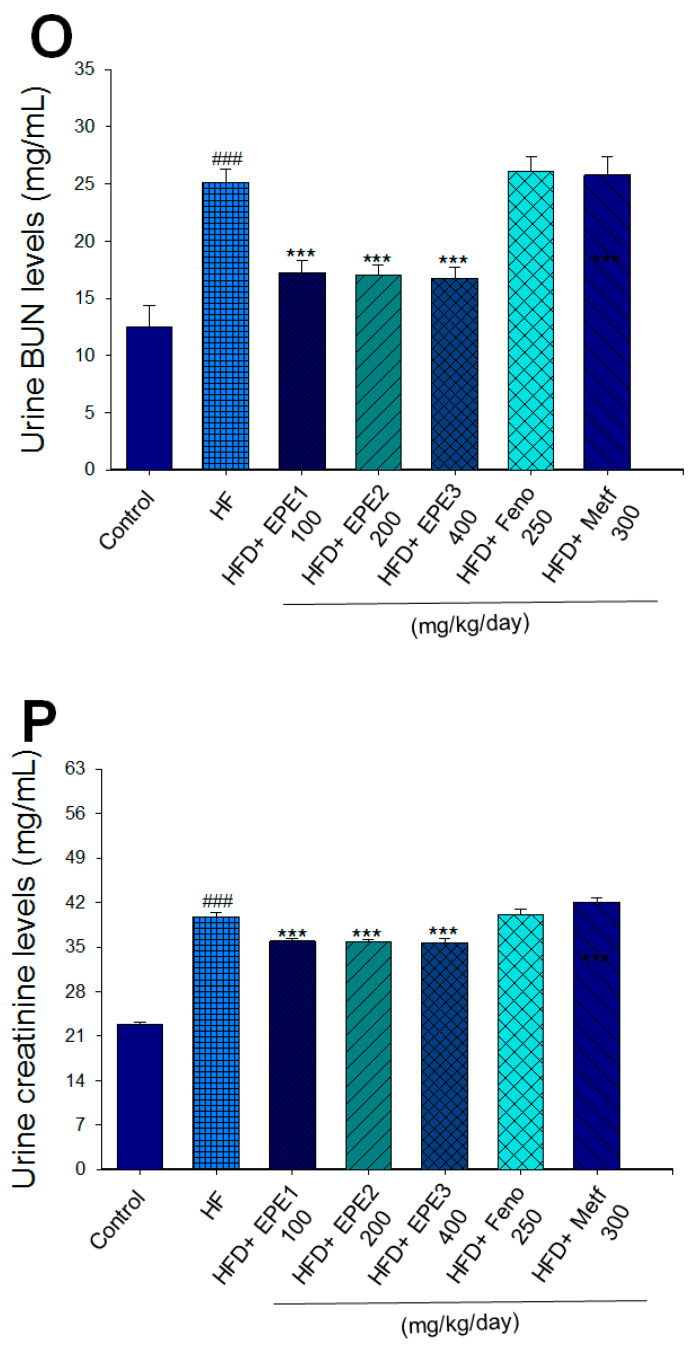

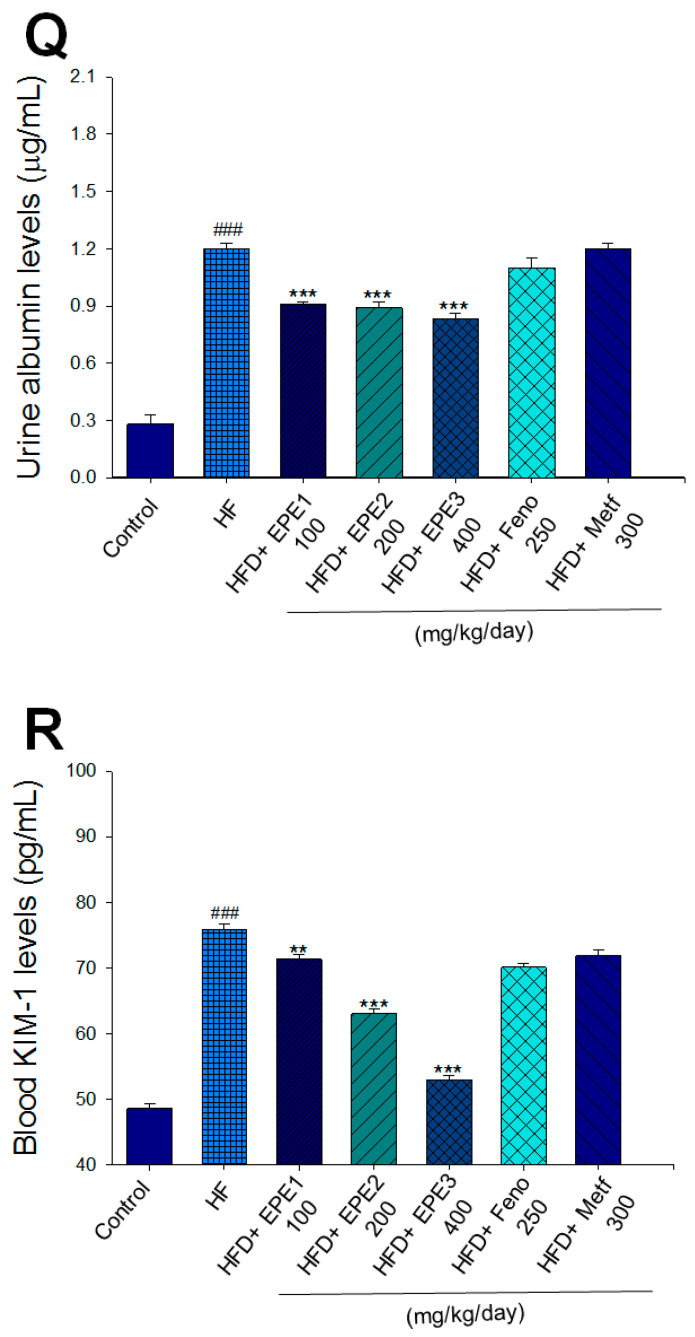

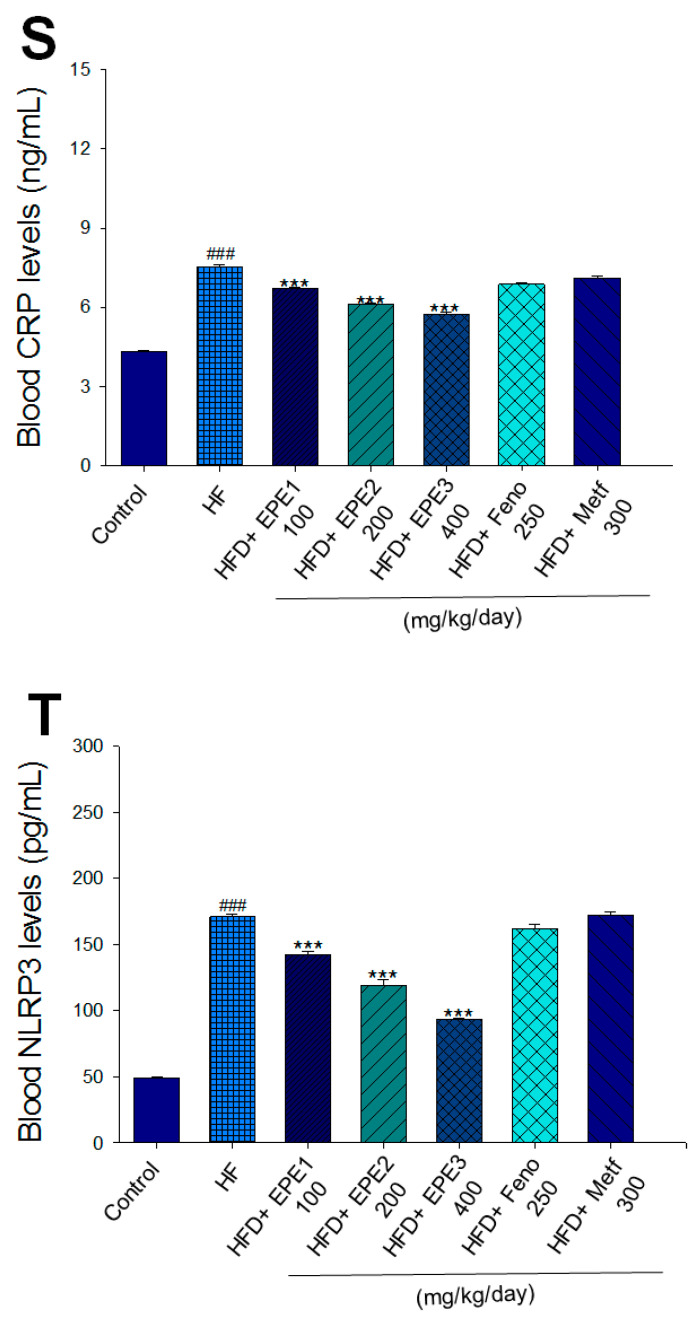
Effects of ethyl acetate extract of *Phyllanthus emblica* L. (EPE) on (**A**) final body weight; (**B**) body weight gain over 4-week treatment; (**C**) absolute fat tissue weight; (**D**) absolute weights of spleen and pancreas; (**E**) blood glucose levels; (**F**) blood glycated hemoglobin (HbA1_C_) levels; (**G**) triglyceride levels; (**H**) total cholesterol levels; (**I**) insulin levels; (**J**) leptin levels; (**K**) adiponectin levels; (**L**) free fatty acid levels; (**M**) blood BUN levels; (**N**) plasma creatinine levels; (**O**) urine BUN levels; (**P**) urine creatinine levels; (**Q**) urine albumin levels; (**R**) blood kidney injury molecule-1 (KIM-1) levels; (**S**) blood CRP levels; and (**T**) blood NLRP3 levels in high-fat diet (HFD)-induced diabetic mice. ^#^ *p* < 0.05, ^##^ *p* < 0.01, and ^###^ *p* < 0.001 compared with the control (CON) group; * *p* < 0.05, ** *p* < 0.01, and *** *p* < 0.001 compared with the high-fat diet (HFD) plus vehicle (distilled water) (HF) group. All values are means ± SE (*n* = 8 per group). The extracts of *Phyllanthus emblica* L. (EPE), EPE1, 100, EPE2, 200, EPE3, 400 mg/kg body weight; Feno, fenofibrate (250 mg/kg body weight); Meft, metformin (300 mg/kg body weight). EWAT, epididymal white adipose tissue; RWAT, retroperioneal white adipose tissue; MWAT, mesenteric white adipose tissue.

As shown in Figure 3A, the HF mice had a greater final body weight than CON mice (*p* < 0.001); no difference exists in body weight at the end among the EPE1-, EPE2-, EPE3-, Feno-, and Metf-HFD mice and the HF group. The body weight gain of HF mice had enhanced relative to CON mice. EPE3, Feno, or Metf in HFD mice lower weight gain in comparison to that of HF mice (Figure 3B). Figure 3B shows that the body weight gain of EPE-treated HFD mice was lower than that of control (not treated and subjected to low fat diet) mice. This means that the extract has weight loss properties like those already observed for fenofibrate and metformin. HF mice had less diet than CON mice. The weights of retroperitoneal, epididymal, visceral fat, or mesenteric white adipose tissue in HF mice were higher than in CON mice (Figure 3C). Treatment with EPE1, EPE2, EPE3, Feno, and Metf exhibited reduced EWAT and visceral fat weights; Feno-treated HFD mice suppressed RWAT weights. Treatment with EPE3 and Feno displayed a decrease in absolute weights of MWAT in comparison to HF mice (Figure 3C). Treatment with Feno or Metf enhanced the hepatic or skeletal muscular weights in comparison to HF mice (Figure 3D).

#### 3.2.2. Blood Glucose, Biochemical Parameters, and Urine Analysis

The HF mice exert higher blood HbA1_C_ and glucose levels than those of CON mice (Figure 3E,F). Treatment with EPE1, EPE2, EPE3, and Metf in HFD mice lowered blood glucose and HbA1_C_ levels (Figure 3E,F). HF mice exerted higher circulating TG and TC concentrations than CON mice (Figure 3G,H). Treatment with EPE1, EPE2, EPE3, Feno, or Metf in HFD mice dramatically lowered circulating TG and TC concentrations when compared with the HF group (Figure 3G,H). HF mice exhibited a dramatic increase in blood insulin or leptin levels but a decrease in adiponectin concentrations relative to CON mice (Figure 3I–K). EPE1, EPE2, EPE3, Feno, or Metf lowered blood insulin and leptin concentrations (Figure 3I,J). EPE1, EPE2, EPE3, Feno, or Metf treatment in HFD mice exhibited higher adiponectin levels than in HF mice (Figure 3K). HF mice increased plasma FFA concentrations as compared with those in CON mice. EPE1, EPE2, EPE3, or Feno in HFD mice lowered FFA concentrations compared with levels in HF mice (Figure 3L).

HF mice exerted higher circulating BUN and creatinine concentrations than levels in CON mice. Treatment with EPE1, EPE2, or EPE3 in HFD mice decreased blood BUN and creatinine levels (Figure 3M,N). The HF mice displayed an increase in urine BUN, creatinine, or albumin levels as compared with CON mice. Treatment with EPE1, EPE2, or EPE3 in HFD mice had decreased urine BUN, creatinine, or albumin levels (Figure 3O–Q). The HF mice displayed higher blood KIM-1, CRP, or NLRP3 levels than the CON mice. Treatment with EPE1, EPE2, or EPE3 in HFD mice lowered blood KIM-1, CRP, or NLRP3 levels in comparison to those of HF mice (Figure 3R–T).

#### 3.2.3. Histopathology Examination

**Figure 4:**
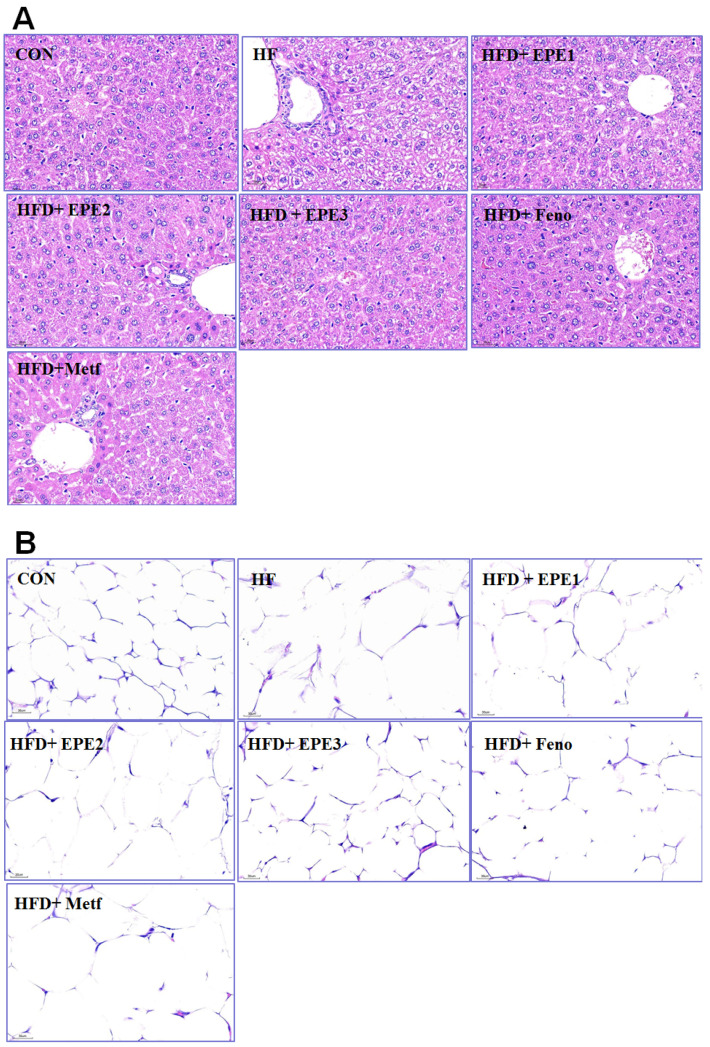
Histology examinations on (**A**) liver tissues (at 400×) and (**B**) white adipose tissues of mice (at 400×) in the control (CON), high-fat diet (HFD) plus vehicle (distilled water) (HF), HFD + EPE1, HFD + EPE2, HFD + EPE3, HFD + fenofibrate (Feno), or HFD + metformin (Metf) groups (*n* = 8 per group) by hematoxylin and eosin staining. The ethyl acetate extract of *Phyllanthus emblica* L. (EPE), EPE1, 100, EPE2, 200, EPE3, 400 mg/kg body weight; Feno, fenofibrate (250 mg/kg body weight); Metf, metformin (300 mg/kg body weight).

HFD feeding developed the hepatocytes ballooning (mean score, 2.3 ± 0.2) as compared with CON mice (0) within the livers. Treatment with EPE1 (1.2 ± 0.2), EPE2 (1.0 ± 0.2), EPE3 (0.7 ± 0.1), Feno (0.6 ± 0.1), and Metf (0.8 ± 0.2) in HFD mice reduced ballooning relatively to HF mice. HFD-feeding progressed a hepatic ballooning degeneration in HF mice in comparison to those of CON mice. Ballooning degeneration is a form of death of the hepatic parenchymal cell and its nucleolus is squeezed into the other side, and it was owing to glycogen accumulation within the nucleus. HFD developed obesity and insulin resistance. Insulin levels influenced the storage of hepatic glycogen. EPE1-, EPE2-, EPE3-, Feno-, or Meft-treated mice significantly decreased the degree of ballooning degeneration (Figure 4A).

The appearance of adipocyte showed a string-like cytosol surrounding a vacuole, and it is owing to it being embedded in paraffin resembling immersed in lipid solvents and could remove all the fats. The unobvious nucleus (N) could be carefully observed on the other side of the cells. HFD-feeding developed hypertrophy of the adipocytes in comparison to CON mice in epididymal WAT. The average area of adipocytes in HF mice is larger than that of CON mice. Treatment with EPE2, EPE3, or Feno reduced adipocyte hypertrophy in comparison to those of HF mice (Figure 4B).

#### 3.2.4. Western Blotting Analysis for Target Gene Expressions

**Figure 5:**
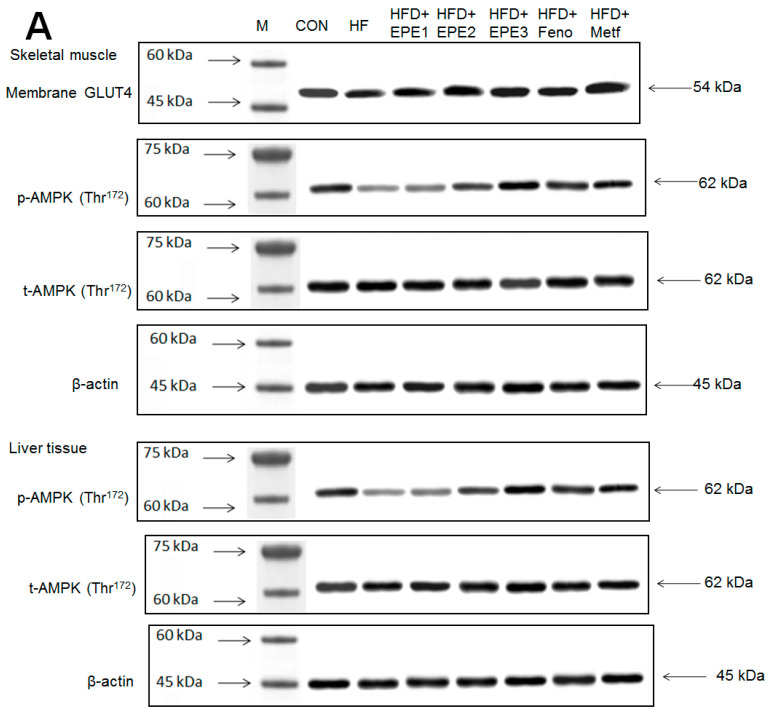

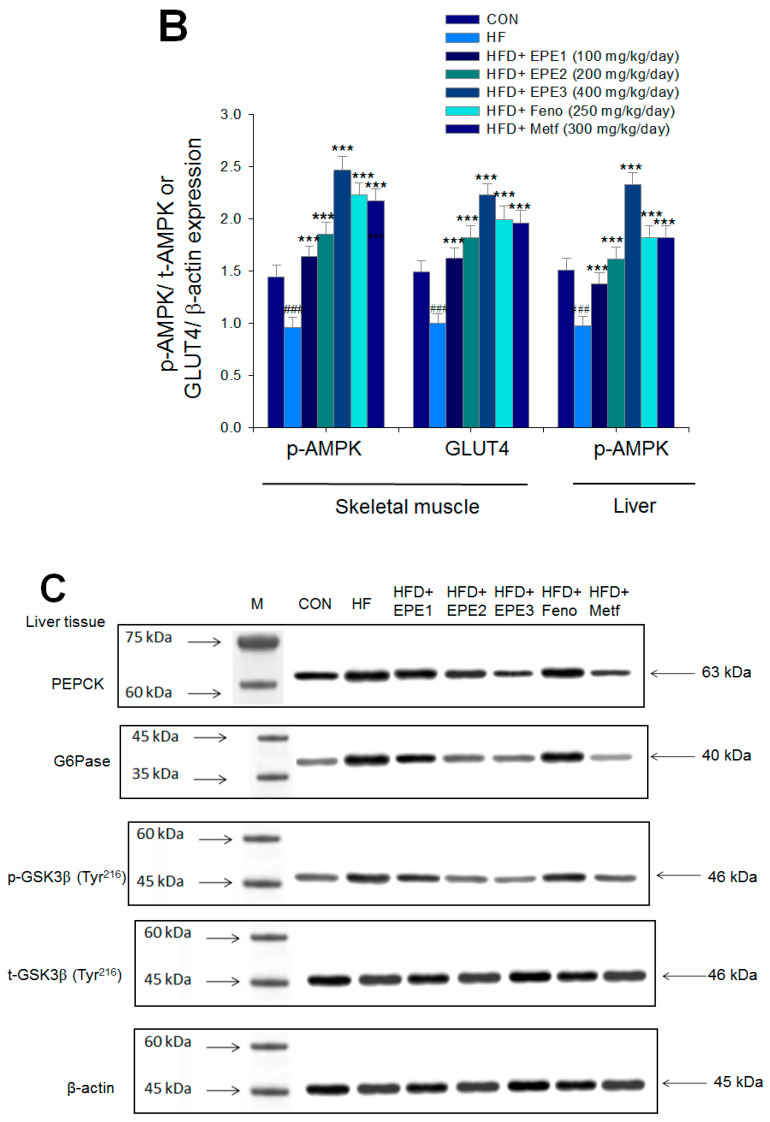

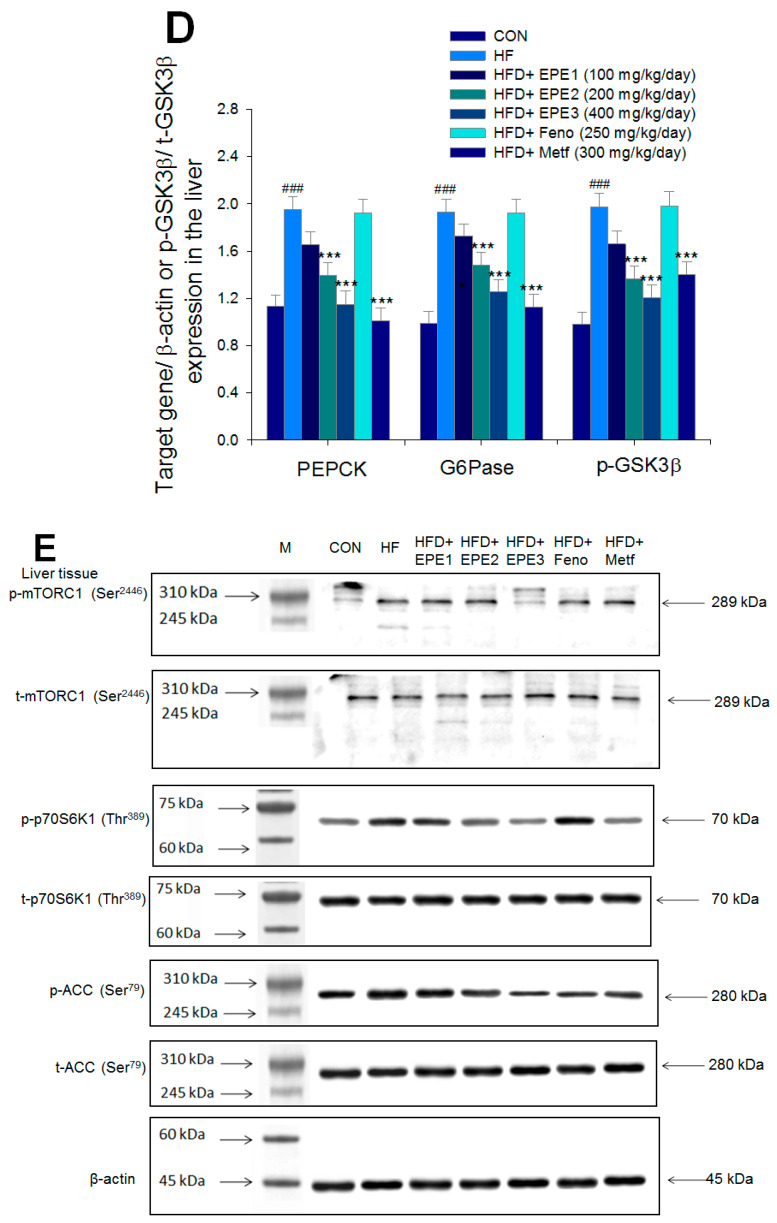

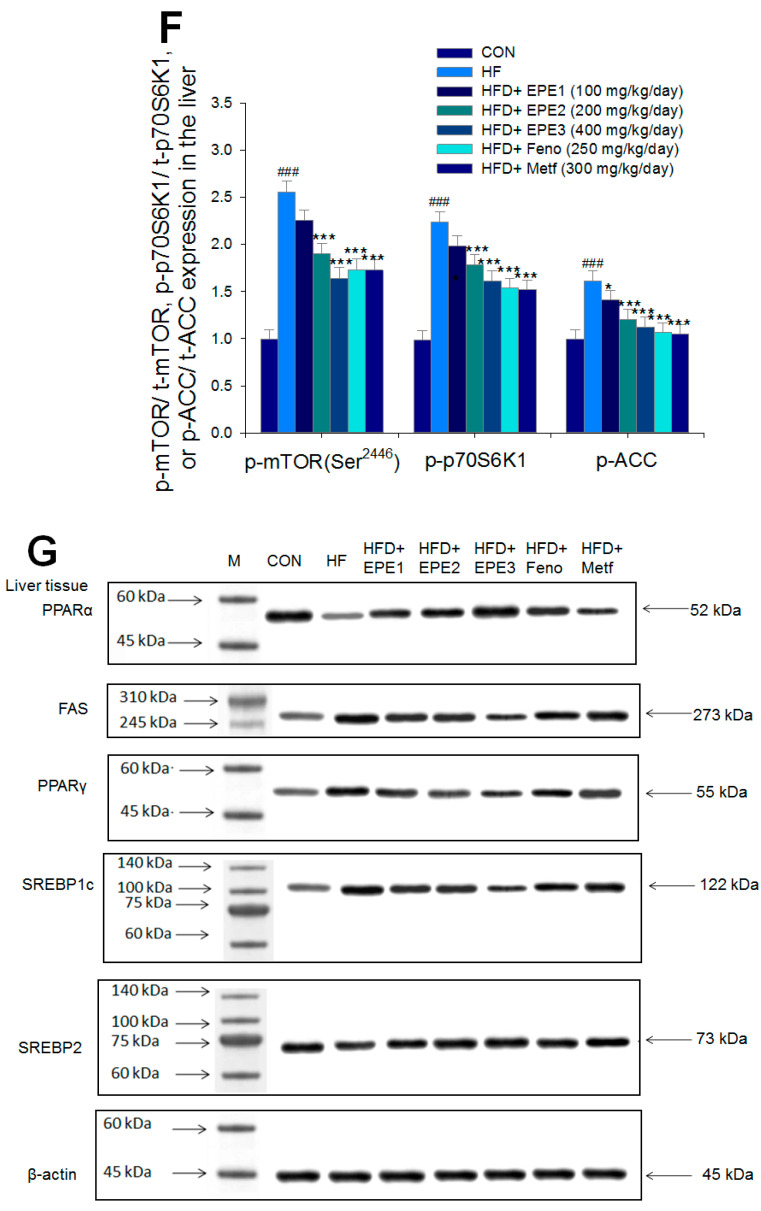

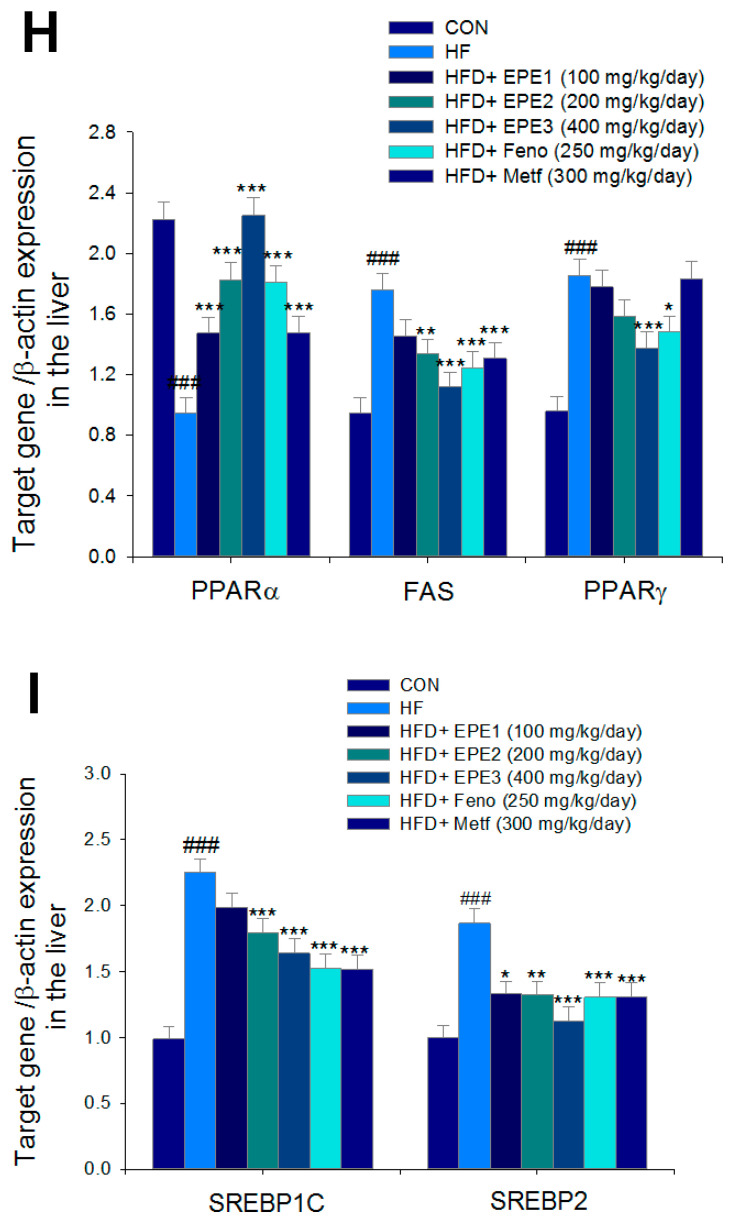

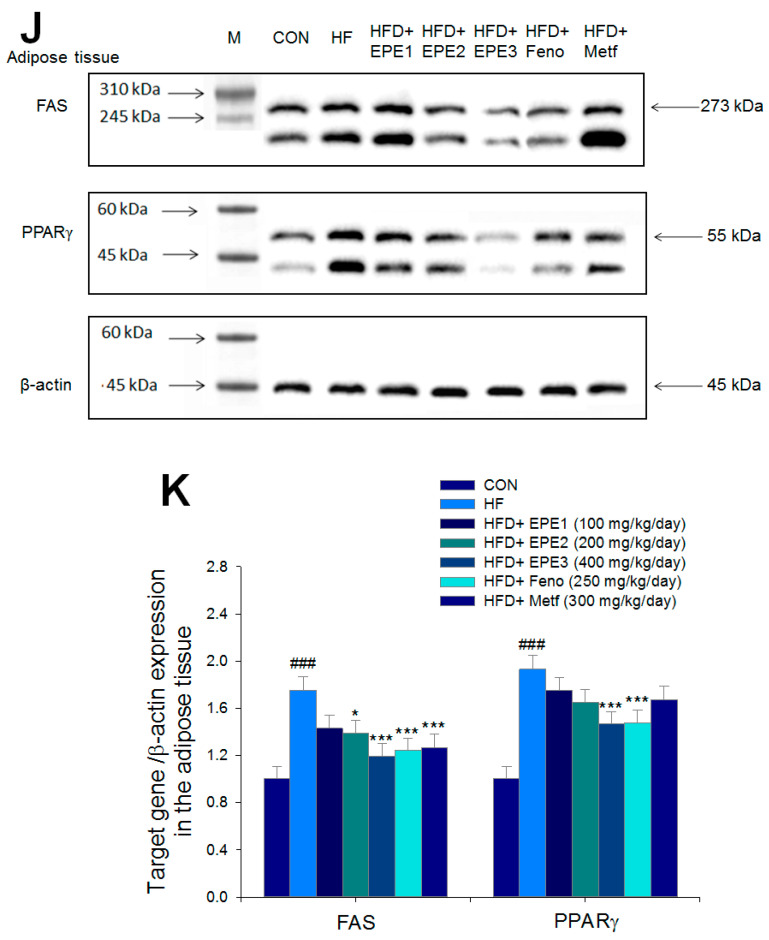

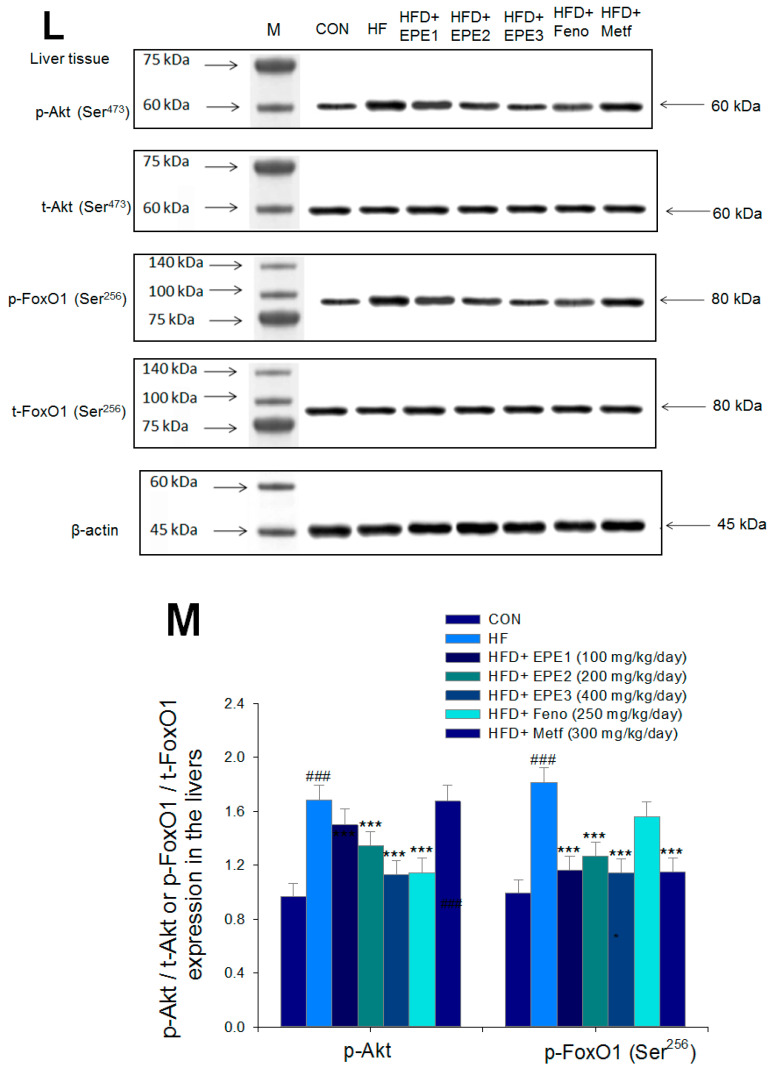
The expression levels of (**A**,**B**) membrane GLUT4, p-AMPK (Thr^172^)/t-AMPK, (**C**,**D**) PEPCK, G6Pase, p-GSK3β/t-GSK3β, (**E**,**F**) p-mTORC1/t-mTORC1, p-p70S6K1/t-p70S6K1, p-ACC/t-ACC, (**G**–**K**) PPARα, FAS, PPARγ, SREBP1_C_, SREBP2 and (**L**,**M**) p-Akt (Ser^473^)/t-Akt (Ser^473^), and p-FoxO1 (Ser^256^)/t-FoxO1 (Ser^256^) in the skeletal muscles, livers, or adipose tissues of high-fat diet (HFD)-induced diabetic mice by oral gavage ethyl acetate extract of *Phyllanthus emblica* L. (EPE). (**A**,**C**,**E**,**G**,**J**,**L**) representative image; (**B**,**D**,**F**,**H**,**I**,**K**,**M**) quantification of the p-AMPK to t-AMPK, p-GSK3β to t-GSK3β, p-mTORC1 to t-mTORC1, p-p70S6K1 to t-p70S6K1, p-ACC to t-ACC p-Akt (Ser^473^) to t- Akt (Ser^473^), and p-FoxO1 (Ser^256^) to t-FoxO1 (Ser^256^). Protein was separated by 12% SDS-PAGE detected by Western blot. ^###^ *p* < 0.001 compared with the control (CON) group; * *p* < 0.05, ** *p* < 0.01, *** *p* < 0.001 compared with the high-fat diet (HFD) plus vehicle (distilled water) (HF) group. All values are means ± SE (*n* = 8 per group). EPE1, EPE2, and EPE3, ethyl acetate extract of *Phyllanthus emblica* L. (EPE) (EPE1, EPE2, and EPE3, 100, 200, and 400 mg/kg body weight, resp.); fenofibrate (Feno; 250 mg/kg body weight); metformin (Metf, 300 mg/kg body weight).

Figure 5A,B showed that the HF group exerted lower skeletal muscular membrane GLUT4 expressions and AMPK phosphorylation expressions both in muscles and livers than CON mice. EPE1, EPE2, EPE3, Feno, or Metf treatment in HFD mice elevated the skeletal muscular membrane GLUT4 expressions but also enhanced the AMPK phosphorylation expressions in both the muscles and the livers in comparison to HF mice.

Figure 5C,D showed that HF mice displayed higher hepatic PEPCK, G6Pase, or p-GSK3β (Tyr^216^)/t-GSK3β (Tyr^216^) expressions than levels in CON mice. Treatment with EPE2, EPE3, or Metf in HFD mice had reduced expression levels of PEPCK, G6Pase, and p-GSK3β (Tyr^216^)/t-GSK3β (Tyr^216^).

Figure 5E,F showed that HF mice had a higher p-mTOR (Ser^2446^)/t-mTOR (Ser^2446^), p-p70S6K1/t-p70S6K1, and p-ACC/t-ACC expressions in the liver tissues than CON mice. Treatment with EPE2, EPE3, Feno, or Metf in HFD mice reduced the hepatic p-mTOR (Ser^2446^)/t-mTOR (Ser^2446^) and p-p70S6K1/t-p70S6K1 expressions. EPE1, EPE2, EPE3, Feno, or Metf treatment in HFD mice lowered hepatic p-ACC/t-ACC expressions in comparison to expressions in HF mice.

Figure 5G,H showed that HF mice lowered PPARα expressions and enhanced hepatic FAS, PPARγ, SREBP2, and SREBP1_C_ expressions as compared with CON mice. EPE, Feno, or Metf treatment in HFD mice increased hepatic PPARα expressions. Moreover, treatment with EPE2, EPE3, Feno, or Metf in HFD mice had reduced expression of FAS, whereas EPE3 and Feno lowered PPARγ expressions in livers as compared with HF mice.

Figure 5G–I showed that treatment with EPE2, EPE3, Feno, or Metf in HFD mice lowered SREBP1_C_ expressions. Treatment with EPE, Feno, or Metf in HFD mice lowered SREBP2 expressions as compared with HF mice.

Figure 5J,K showed that HF mice elevated FAS and PPARγ expressions in adipose tissues compared with CON mice. Treatment with EPE2, EPE3, Feno, or Metf in HFD mice decreased FAS expressions, and EPE3 or Feno lowered PPARγ expressions in adipose tissues in comparison to levels in HF mice.

Figure 5L,M showed that HF mice had higher hepatic p-FOXO1/t-FOXO1 and p-Akt (Ser^473^)/t-Akt (Ser^473^) expressions than CON mice. Treatment with EPE2, EPE3, or Feno in HFD mice lowered p-Akt (Ser^473^)/t-Akt (Ser^473^) expressions in livers as compared with HF mice. EPE or Metf treatment in HFD mice decreased p-FOXO1/t-FOXO1 expressions in comparison to levels in HF mice.

### 3.3. Effects of Re-Fractions of EPE on AMPK Activation by Western Blotting

Following the animal study, we were interested in finding out the marker active ingredient of EPE and therefore both Western blotting and HPLC analyses were adapted. At the molecular levels, we assessed the p-AMPK/t-AMPK expressions (representing AMPK activation) that are responsible for both the antihyperglycemic and antihyperlipidemic activity.

**Figure 6:**
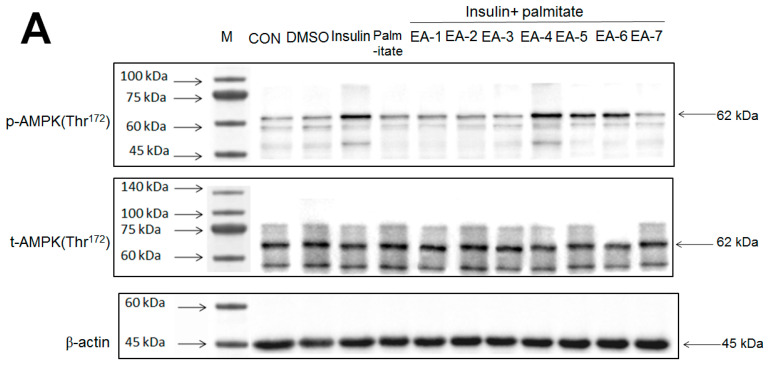

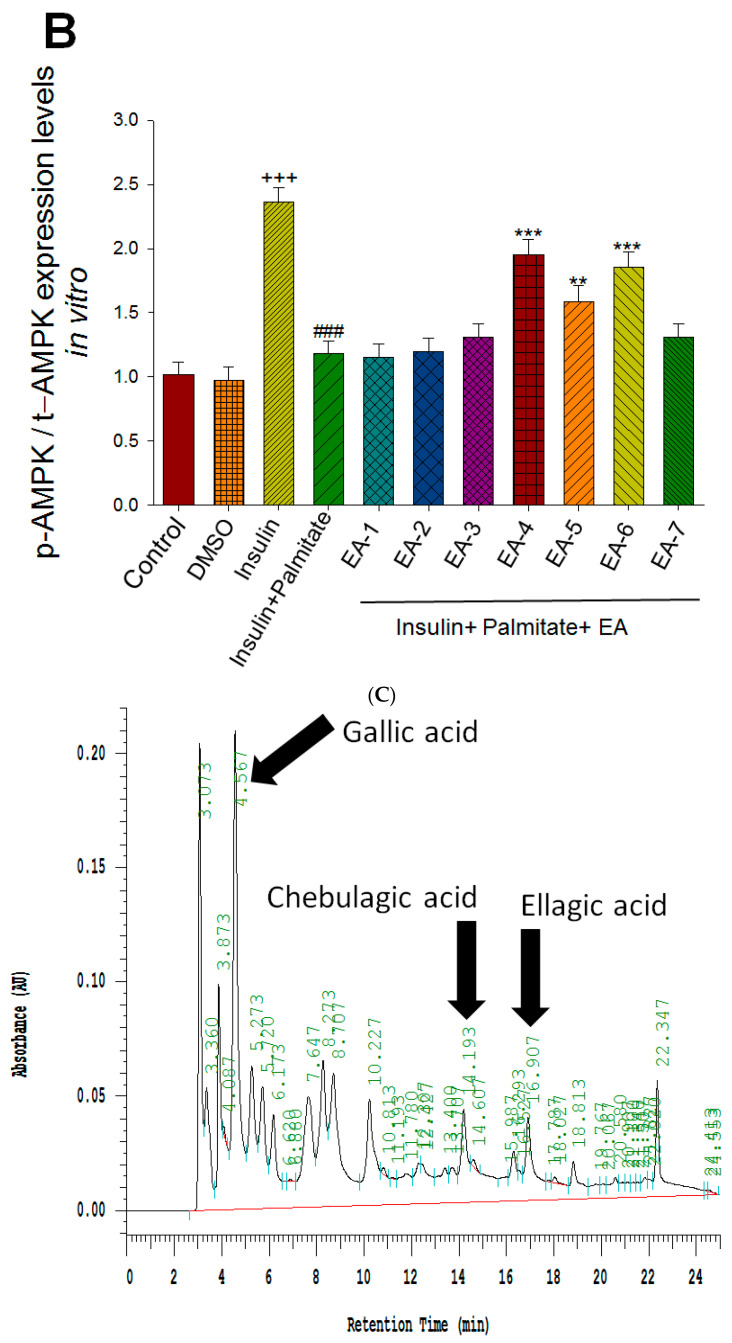

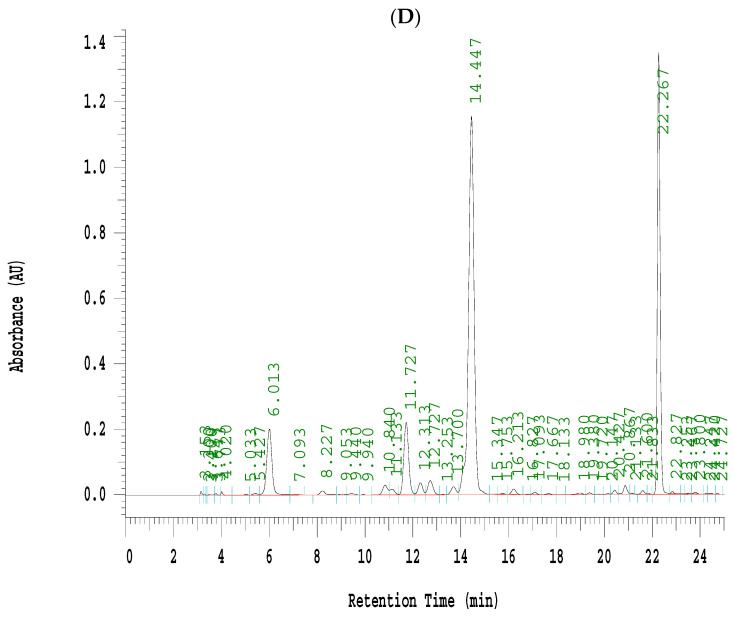
Effects of seven fractions of EPE (EA) on expression levels of phospho-AMPK/total-AMPK in C2C12 myoblasts cells by Western blotting analyses. C2C12 skeletal myoblast cells were treated with seven fractions and equal amounts of lysates were resolved by SDS-PAGE and blotted for phospho-AMPK (Thr^172^). (**A**,**B**) Effects of 7 fractions of ethyl acetate extract of *Phyllanthus emblica* L. (EtOAc soluble fractions) (EA-1, EA-2, EA-3, EA-A, EA-5, EA-6, and EA-7) on expression levels of phospho-AMPK (Thr^172^) in C2C12 myoblasts cells by Western blotting analysis. C2C12 skeletal myoblast cells were treated with 7 fractions as described in the experimental procedures and equal amounts of lysates were resolved by SDS-PAGE and blotted for phospho-AMPK (Thr^172^). (**A**) Representative blots for 7 fractions in C2C12 myoblasts cells; (**B**) quantification of the expression levels of the ratio of phospho-AMPK to total-AMPK. All values are means ± S.E. ^+++^ *p* < 0.001 compared with the control group; ^###^ *p* < 0.001 compared with the insulin group ** *p* < 0.01, *** *p* < 0.001 compared with the palmitate+ insulin group. (**C**,**D**) High-performance liquid chromatography analysis of (**C**) 2500 ppm ethyl acetate of *Phyllanthus emblica* L. (EPE), (**D**) 10.3 mg/5 mL EA-4 of ethyl acetate of *Phyllanthus emblica* L. [55].

Figure 6A,B showed that AMPK phosphorylation expressions in tC2C12 myotubes were markedly enhanced by insulin relative to the control group; these increases were prevented by palmitate (in comparison to the insulin group); and then restored by EA-4, EA-5, and EA-6 compared with the insulin + palmitate group. The Western blotting data have shown that EA-4 exhibits the best activity of AMPK activation among seven re-fractions.

### 3.4. EA-4 Re-Fraction of EPE HPLC Analysis

Evidence indicated that EPE contained a variety of phenolic compounds including chebulagic acid (6.44%), gallic acid (3.28%), and ellagic acid (2.23%) [34]. This study selected of EA-4 sample due to EA-4 displaying the best activity of p-AMPK phosphorylation expressions (Figure 6A,B). Coincidently, the marker component of EA-4 has been investigated in our recent studies by db/db mouse model, which is regarded as a murine model to develop diabetic nephropathy and T2DM [55]. Figure 6C,D was our recent publication [55] and showed that the marker of phenolic compounds is chebulagic acid that existed in EA-4 (54.4%) [55].

## 4. Discussion

The model of HFD feeding is recognized as a means to induce diabetes in mice, and these mice display metabolic disorders similar to human type 2 diabetes. Therefore, we chose this model to investigate how EPE affects hyperglycemia and hyperlipidemia. Our results showed that mice on a long-period HFD exhibited hyperglycemia, hypertriglyceridemia, hypercholesterolemia, hyperinsulinemia, and higher levels of blood HbA1_C_, FFA, BUN, and plasma creatinine than those of CON mice, and these consequences were in accordance with previous studies [40,41]. Following the administration of EPE to HFD-fed mice, hyperglycemia and hyperinsulinemia were effectively controlled by dramatically decreasing blood glucose levels and insulin concentrations; furthermore, blood glycosylated HbA1c concentrations, a long-term control marker, were reduced. Our study results indicated that administration of EPE to HFD-fed mice displayed activity similar to that of an insulin sensitizer to improve peripheral insulin resistance, including in adipose and liver tissue, and skeletal muscles.

The histology results indicated that treatment with EPE in HFD mice had reduced adipose tissue hypertrophy and hepatic ballooning compared to those of vehicle-treated HFD mice. EPE treatment decreased visceral fat amounts, which is involved in insulin resistance, implying EPE improved peripheral insulin resistance. This study indicated that HFD-fed mice displayed hypertriglyceridemia and hypercholesterolemia, as a previous study described [56]. Treatment with EPE in HFD mice lowered circulating lipid concentrations, including a reduction in plasma TG, TC, and FFA concentrations, implying that EPE displayed antihyperlipidemic activity in HFD mice. Collectively, EPE displayed preventive activity against diabetes mellitus and hyperlipidemia in HFD-induced T2DM mice.

In this study, the efficacy and mechanism of EPE on AMPK activation and the membrane GLUT4 protein expression were examined in HFD-induced T2DM mice in comparison with Feno and Metf treatments. The target gene expressions related to antidiabetic and antihyperlipidemic mechanisms in the peripheral tissues were also examined. To explore the mechanism responsible for the antidiabetic activity of EPE, we investigated whether EPE affects membrane GLUT4 expression. These results presented here indicated that EPE treatment led to increasing membrane GLUT4 expressions in skeletal muscles in HFD mice, implying that EPE caused a rise in glucose uptake shifting blood toward cellular sites, thus resulting in a reduction in blood glucose concentrations.

To clarify whether EPE could display a rise in AMPK activation, which is significant because AMPK activation is the key promoter of GLUT4 translocation in physical activity or concerning several antidiabetic drugs, including the clinic drug, metformin [4]. EPE significantly elevated the p-AMPK/t-AMPK expressions compared with expression in the HF group and was quite similar to those of Metf mice, implying that enhanced p-AMPK/t-AMPK expressions are partly accountable for the promotion of GLUT4 translocation by EPE.

Our results showing high-fat induction display a rise in hepatic PEPCK and G6Pase expressions are consistent with those of Berg et al. (2001), which showed the livers of diabetic animals display a marked increase in the G6Pase activity [46]. Evidence indicated that AMPK activation produced a decrease in gluconeogenic PEPCK and G6Pase expressions in liver tissues [57].

FOXO1 will activate the transcription of phosphoenolpyruvate carboxykinase [58]. While hepatic FOXO1 is expressed, fasted glucose concentrations are enhanced [59]. In contrast, hepatic-specific FOXO1 knock-out mice will progress faster with low blood sugar [60]. The mouse could keep glucose homeostasis by fasting and feeding while without both Akt and FOXO1 [61]. Previous studies show an opportunity for novel therapies for diabetes mellitus [12] and the relationship among FOXO1, gluconeogenesis, AMPK activation, PEPCK, G6Pase, and Akt [13,58,59,60,61]. The present study showed that treatment with EPE2, EPE3, and Metf in HFD mice exhibited a reduction in hepatic p-FOXO1/t-FOXO1 expressions and lowered the gluconeogenic G6Pase and PEPCK expressions. This response implied that EPE could improve hyperglycemic status possibly resulting from AMPK activation and/or the regulation of FOXO1 phosphorylation, thereby affecting PEPCK and G6Pase expressions in liver tissues by concomitant hepatic suppression of gluconeogenesis.

Collectively, the study outcome indicated that the underlying molecular mechanisms of EPE’s antihyperglycemic activity are that EPE promotes a rise in the skeletal muscular GLUT4 expressions to elevate glucose uptake shifting the blood toward the cellular position and enhancing p-AMPK/t-AMPK expressions in both liver tissues and skeletal muscles convergently to decrease blood glucose concentrations. Additionally, regarding previous studies [9,11,12], the experiment showed that EPE-treated HFD mice have reduced PEPCK and G6Pase expression in liver tissues to inhibit hepatic glucose production and increase phosphorylated Akt levels while reducing phosphorylated GSK3β, rendering glycogen synthesis active, with a consequent enhanced potential for glucose uptake and glycogenesis, thus contributing to an antidiabetic effect.

To clarify how EPE acts on lipid catabolism and its underlying molecular mechanisms such as the target FAS, SREBP-1c, and -2, PPARγ, and PPARα expressions. According to previous studies showing that numerous targeted genes [22,23,24,25,26,27], our experiment indicated that treatment with EPE in HFD mice markedly increased PPARα while decreasing the SREBP1c, PPARγ, and FAS expressions, suggesting that EPE could modulate these enzymes gene expressions by decreases in the SREBP1c, PPARγ, and FAS expressions thus ameliorating lipid metabolic abnormalities.

Evidence has shown that leptin activates AMPK, inactivates ACC, and promotes the balance of FA oxidation [62]. The glucose-induced FAS expressions were reduced by AMPK activation, suggesting that EPE could increase direct and/or leptin-mediated AMPK activation [63]. Evidence [9] indicated that metformin downregulated SREBP1c expression to suppress the expression level of FAS through the activation of AMPK. Thus, there are two possibilities EPE regulated these genes or/and down-regulated gene expressions by AMPK activation.

Adipocytokine plays a core part in insulin resistance. Blood adiponectin levels are lowered in insulin-resistant status and obesity [63]. The discovery of enhanced adiponectin expressions brought many benefits to insulin resistance [64]. Evidence implied that administration of the globular domain of adiponectin enhanced AMPK activation and glucose uptake [65]. Another evidence indicated that leptin activates AMPK and then with relation to the improvement of FA oxidation and the inhibition of triacylglycerol accumulation [66]. According to previous studies [63,64] showing the relationship between adiponectin concentration level and insulin resistance, our findings revealed that the EPE treatment in HFD mice enhanced adiponectin concentrations while decreasing leptin levels (Figure 3J,K) and reducing target gene expression (including PPARγ and FAS) in adipose tissues (Figure 5J). EPE could have beneficial value in adipocyte function modulation to ameliorate insulin resistance.

To clarify the effects of EPE on adipocytokines, we examine blood levels of adiponectin and leptin. According to previous evidence [65,66], the AMPK activation by EPE is due to being involved in leptin and/or adiponectin secretion, and EPE could directly increase activation of AMPK or regulate adiponectin and/or leptin secretion to induce activation of AMPK.

Enhanced mTOR signaling was demonstrated in T2DM [28,29,30,31]. Furthermore, AMPK action stimulated by metformin results in the suppression of mTORC1 [67] in a manner of TSC1/2-dependent [68]. Intracellular energy amounts modulate the activity of mTORC1 through TSC or Raptor responding to AMPK or through Rag GTPase in a manner of AMPK-independent [69,70]. Based on the relationship among mTORC1, S6K1, and AMPK activation [28,29,30,31,67,68,69,70], our findings indicated that EPE or Metf treatment in HFD mice reduced the mTORC1 and S6K1 phosphorylation and decreased AMPK activation in the liver, implying that EPE displays antihyperlipidemic activity by AMPK activity enhancement is dependent or independent and inhibiting mTORC1 activity.

A total of 75% of T2DM patients are likely to progress diabetic nephropathy if these individuals suffered long-term term for above 35 years. To clarify the effects of EPE on renal dysfunction, we examined blood inflammatory cytokine and renal function assessments. Our presented results are similar to those of previous studies [40,41] indicating that HFD-induced diabetic mice exhibited higher levels of blood HbA1_C_, FFA, BUN, and plasma creatinine than those of CON mice. Treatment with EPE in HFD mice not only lowered circulating lipid concentrations, including a reduction in plasma TG, TC, and FFA concentrations but also reduced BUN and plasma creatinine levels in both urine and blood while also lowering urine albumin levels in HFD-induced diabetic mice, implying that EPE displayed antihyperlipidemic activity in HFD-fed mice, and EPE possesses protective activity on renal dysfunction. The chronic hyperlipidemic state led to excess renal lipid accumulation to pro-inflammatory secretion, producing cellular injury and renal dysfunction [40,71]. EPE ameliorated renal dysfunction (including urinary albumin excretion) in part by the inhibition of inflammatory factors in HFD-induced diabetic mice, implying EPE exhibits a protective effect from renal dysfunction by anti-inflammation in Type 2 diabetic HFD-fed mice. Renal inflammation and subsequent fibrosis play important roles in leading to end-stage DN. EPE is demonstrated to ameliorate diabetic nephropathy by inhibiting inflammation and suppressing fibrosis and within the kidney in db/db mice as our recent report [55]. All of these results implied that EPE could alleviate diabetic nephropathy by suppressing inflammation and renal fibrosis in these two different Type 2 diabetes animal models.

After the mice treatments, the research work was engaged in finding out the marker component of EPE responsible for improving insulin resistance using seven fractions of EPE. Evidence indicated that the chebulagic acid of three polyphenol components exerts antioxidant [72]. In in vitro tests, Chebulagic acid was demonstrated to be a powerful α-glucosidase blocker, implying it has promise in the treatment of T2DM [73]. Chebulagic acid was demonstrated to display a critical part in glucose uptake in vitro but also acts like a partial PPARγ agonist which could be exploited in the management of T2DM [74]. Chebulagic acid has an antiangiogenic effect associated with VEGFR2 signaling pathway inhibition, and this is important in the context of inflammation as a risk factor for tumor development, co-dependence of inflammation and angiogenesis, and the anti-inflammatory actions of chebulagic acid [75]. Chebulagic acid and chebulinic acid were demonstrated to inhibit TGF-β1 induced fibrotic changes in the chorio-retinal endothelial cells and as potential adjuvants in the management of diabetic retinopathy [76]. EPE is demonstrated to protect from diabetic nephropathy by its maker component chebulagic acid with an antiangiogenic effect involved in VEGF inhibition and anti-inflammatory in db/db mice [55].

Our findings from the Western blotting analysis (Figure 6) have shown that EA-4 refraction of EPE displays the best activity among seven refractions of EPE with respect to the increases in p-AMPK/t-AMPK expressions, implying that the main constituent is chebulagic acid, yielded an extraction percentage of total polyphenol chebulagic acid of >54.4% [55] and that this responsible for the both of antihyperlipidemic and antihyperglycemic activities and the improvement of metabolic disorders such as diabetic renal dysfunction in HFD-induced diabetic mice, which is an animal model of T2DM.

Long-term HFD feeding is known to develop kidney injury (including enhancement in oxidative stress and tissue lipid accumulation) [40]. Our findings showed that one of the main components of EPE is chebulagic acid, which possesses antioxidant activity [72], suggesting that EPE improves renal function in HFD-produced T2DM mice partly due to the antioxidant activity of its main constituent, chebulagic acid, and indirectly decreases lipid depots, and decreases pro-inflammatory cytokine including KIM-1 levels. EPE treatment in HFD mice lowered the BUN and creatinine levels both in urine and blood. Furthermore, treatment with EPE lowered circulating inflammation cytokines concentrations, such as NLRP3, CRP, and KIM-1. The present data indicated that EPE had antihyperglycemic activities but also ameliorated renal dysfunction in HFD mice. An explanation for EPE’s functional activities is that the EPE containing total polyphenol chebulagic acid has the major activities of anti-diabetes mellitus, anti-hyperlipidemia, and indirectly modulating pro-inflammation cytokines, as well as mutual restraint of lipid accumulation among the peripheral tissues including adipose tissues (decreasing lipid depots), liver tissue (increasing metabolism), and skeletal muscles as a consequence of the protective efficacy of EPE from T2DM, insulin resistance, and lipid disorders. Therefore, we found that EPE could have an advantageous effect in HFD mice as a result of producing proof of the capacity of alteration in inflammatory cytokines and amelioration of renal dysfunction.

## 5. Conclusions

**Figure 7:**
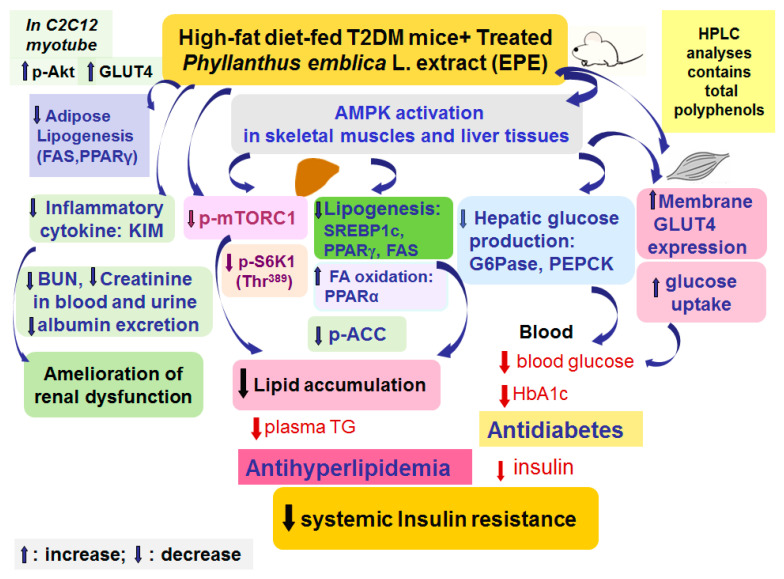
The graphic abstract of ethyl acetate extract of *Phyllanthus emblica* L. (EPE) in high-fat diet (HFD)-induced diabetic mice.

EPE has a beneficial perspective in the treatment of T2DM which is presented by its antihyperglycemic and antihyperlipidemic effects in HFD-induced diabetic animal models (Figure 7). According to our results on blood glycemia and circulating lipids levels, the model of HFD-fed mice displayed hyperglycemia, hypertriglyceridemia, and hypercholesterolemia. Treatment with EPE in HFD mice lowered blood glucose and HbA1_C_ levels. EPE treatment elevated GLUT4 proteins to enhance glucose uptake, while the hepatic PEPCK and G6Pase expressions were reduced to suppress glucose production, resulting in lowering glucose levels. Furthermore, EPE-treated HFD diabetic mice not only had lower creatinine and BUN concentrations in both urine and blood but also had decreased albumin levels, implying that EPE could possess preventive activity on renal function in HFD mice. Moreover, EPE treatment in HFD mice lowered blood triglycerides and total cholesterol levels. EPE treatment increased p-AMPK/AMPK expression levels in both skeletal muscles and the livers but lowered the hepatic p-mTORC1/t-mTORC1 and p-p70S6K1 (Thr^389^)/t-p70S6K1 (Thr^389^) expressions, accompanied by decreased SREBP1_C_, SREBP2, FAS, and PPARγ expressions, but enhanced PPARα expressions, as a consequence of lowering blood TG and TC levels and improving insulin resistance. The present study implied that EPE diminished visceral obesity and hyperlipidemia and improves insulin resistance by lowering mTOR/S6K1 signaling proteins but elevating insulin sensitivity both in peripheral tissues.

## Data Availability

All data used to support the findings of this study are available from the corresponding author, Chun-Ching Shih, upon reasonable request. Corresponding author’s email: ccshih@ctust.edu.tw.

## Abbreviations

ACC, acetyl-CoA carboxylase; AMPK, AMP-activated protein kinase; CON, control; FAS, fatty acid synthase; Feno, fenofibrate; FFA, free fatty acid; FOXO1, forkhead transcription factor forkhead box O1; GSK3β, glycogen synthase kinase β; GLUT4, glucose transporter 4; HbA1c, glycated hemoglobin; HFD, high-fat-diet; Metf, metformin; mTOR, mammalian target of rapamycin; mTORC1, mammalian target of rapamycin complex C1; S6K1, S6 kinase 1; WAT, white adipose tissue.
